# Bone microarchitecture and strength assessed by HRpQCT in individuals with type 2 diabetes and prediabetes: the Maastricht study

**DOI:** 10.1093/jbmrpl/ziae086

**Published:** 2024-07-03

**Authors:** Veerle Van Hulten, Cindy Sarodnik, Johanna H M Driessen, Rikke Viggers, Nicklas H Rasmussen, Piet P M M Geusens, Nicolaas Schaper, Miranda T Schram, Bastiaan E De Galan, Annemarie Koster, Sandrine P G Bours, Peter Vestergaard, Coen D A Stehouwer, Joop P van den Bergh

**Affiliations:** Department of Clinical Pharmacy and Toxicology, Maastricht University Medical Centre+ (MUMC+), P. Debyelaan 25, 6229 HX, Maastricht, the Netherlands; School of Nutrition and Translational Research in Metabolism (NUTRIM), Maastricht University, Universiteitssingel 50, 6229 ER, Maastricht, the Netherlands; Cardiovascular Research Institute Maastricht (CARIM), Maastricht University, Universiteitssingel 50, 6229 ER, Maastricht, the Netherlands; Division of Rheumatology, Department of Internal Medicine, Maastricht University Medical Centre+ (MUMC+), P. Debyelaan 25, 6229 HX, Maastricht, the Netherlands; Department of Clinical Pharmacy and Toxicology, Maastricht University Medical Centre+ (MUMC+), P. Debyelaan 25, 6229 HX, Maastricht, the Netherlands; Cardiovascular Research Institute Maastricht (CARIM), Maastricht University, Universiteitssingel 50, 6229 ER, Maastricht, the Netherlands; Steno Diabetes Center North Denmark, Aalborg University Hospital, Søndre Skovvej 3E, 9000, Aalborg, Denmark; Steno Diabetes Center North Denmark, Aalborg University Hospital, Søndre Skovvej 3E, 9000, Aalborg, Denmark; Division of Rheumatology, Department of Internal Medicine, Maastricht University Medical Centre+ (MUMC+), P. Debyelaan 25, 6229 HX, Maastricht, the Netherlands; Care and Public Health Research Institute (CAPHRI), Maastricht University, Universiteitssingel 50, 6229 ER, Maastricht, the Netherlands; Biomedical Research Institute, University Hasselt, Agoralaan, 3590, Hasselt, Belgium; Cardiovascular Research Institute Maastricht (CARIM), Maastricht University, Universiteitssingel 50, 6229 ER, Maastricht, the Netherlands; Care and Public Health Research Institute (CAPHRI), Maastricht University, Universiteitssingel 50, 6229 ER, Maastricht, the Netherlands; Department of Internal Medicine, Maastricht University Medical Centre+ (MUMC+), P. Debyelaan 25, 6229 HX, Maastricht, the Netherlands; Cardiovascular Research Institute Maastricht (CARIM), Maastricht University, Universiteitssingel 50, 6229 ER, Maastricht, the Netherlands; Department of Internal Medicine, Maastricht University Medical Centre+ (MUMC+), P. Debyelaan 25, 6229 HX, Maastricht, the Netherlands; Heart and Vascular Center, Maastricht University Medical Center+ (MUMC+), P. Debyelaan 25, 6229 HX, Maastricht, the Netherlands; Cardiovascular Research Institute Maastricht (CARIM), Maastricht University, Universiteitssingel 50, 6229 ER, Maastricht, the Netherlands; Department of Internal Medicine, Maastricht University Medical Centre+ (MUMC+), P. Debyelaan 25, 6229 HX, Maastricht, the Netherlands; Department of Internal Medicine, Radboud University Medical Center, Geert Grooteplein Zuid 10, 6525 GA, Nijmegen, the Netherlands; Care and Public Health Research Institute (CAPHRI), Maastricht University, Universiteitssingel 50, 6229 ER, Maastricht, the Netherlands; Department of Social Medicine, Maastricht University, Universiteitssingel 50, 6229 ER, Maastricht, the Netherlands; Division of Rheumatology, Department of Internal Medicine, Maastricht University Medical Centre+ (MUMC+), P. Debyelaan 25, 6229 HX, Maastricht, the Netherlands; Steno Diabetes Center North Denmark, Aalborg University Hospital, Søndre Skovvej 3E, 9000, Aalborg, Denmark; Steno Diabetes Center North Denmark, Department of Endocrinology, Aalborg University Hospital, Mølleparkvej 4, 9000, Aalborg, Denmark; Cardiovascular Research Institute Maastricht (CARIM), Maastricht University, Universiteitssingel 50, 6229 ER, Maastricht, the Netherlands; Department of Internal Medicine, Maastricht University Medical Centre+ (MUMC+), P. Debyelaan 25, 6229 HX, Maastricht, the Netherlands; School of Nutrition and Translational Research in Metabolism (NUTRIM), Maastricht University, Universiteitssingel 50, 6229 ER, Maastricht, the Netherlands; Division of Rheumatology, Department of Internal Medicine, Maastricht University Medical Centre+ (MUMC+), P. Debyelaan 25, 6229 HX, Maastricht, the Netherlands; Subdivision of Endocrinology, Department of Internal Medicine, VieCuri Medical Center, Tegelseweg 210, 5912 BL, Venlo, the Netherlands

**Keywords:** type 2 diabetes, HRpQCT, bone, fracture, bone microarchitecture

## Abstract

Type 2 diabetes (T2D) is a prevalent disease and has been associated with an increased fracture risk despite normal or even higher areal BMD. The aim of this study was to estimate the association between glucose metabolism status (GMS) and measurements of glycemic control with HRpQCT parameters of bone microarchitecture and strength. Participants of the Maastricht study who underwent an HRpQCT scan at the distal radius and tibia were included. GMS was determined by use of an oral glucose tolerance test and grouped into a normal glucose metabolism (NGM), prediabetes, or T2D. Linear regression models were used, stratified by sex with multiple adjustments. This study incorporated cross-sectional data from 1400 (796 [56.9%] NGM, 228 [16.3%] prediabetes, and 376 [26.9%] T2D) men and 1415 (1014 [71.7%] NGM, 211 [14.9%] prediabetes, and 190 [13.4%] T2D) women. The mean age was 59.8 ± 8.6 and 57.6 ± 9.0 yr for men and women, respectively. After adjustment, T2D was associated with a higher total BMD measured by HRpQCT and cortical thickness, and a smaller total and trabecular area in men and women compared with NGM. In women, T2D was additionally associated with a higher stiffness and failure load at the radius. Results were more pronounced at the distal radius than at the distal tibia. To conclude, these findings suggest that in this cohort of Maastricht study participants, total and trabecular bone area are smaller, but bone microarchitecture, density, and bone strength assessed by HRpQCT are not impaired in individuals with T2D.

## Introduction

Type 2 diabetes (T2D) is a multifaceted disease with a rising prevalence and is characterized by beta cell failure and varying levels of insulin resistance, causing blood glucose levels to rise toward hyperglycemia.^1^ This hyperglycemic state is responsible for numerous negative health consequences, mainly related to the damage of the macro- and microvascular system.^2^ T2D has also been associated with an increased fracture risk, despite normal or even increased areal bone mineral density (aBMD).^3^

Observations of greater fracture risk in T2D suggest that bone quality and fracture risk are not solely determined by BMD. Indeed, the ability of bone to withstand fracture, from now on referred to as bone quality,^4^ largely depends on cortical and trabecular bone microarchitecture, which might be altered in T2D due to impaired blood flow, advanced glycation end product (AGE) accumulation, and adipogenesis.^5–7^

While the DXA scan is most frequently used to estimate fracture risk, it is unable to map the bone micro-architecture and consequently bone quality. However, compartmental bone microarchitecture in the distal radius and tibia can be analyzed by HRpQCT.^8^ Walle et al.^9^ recently meta-analyzed diabetes associated differences in bone structure in 516 T2D participants available from 12 studies. While some studies in this meta-analysis showed impairments in the cortical or trabecular bone compartment,^10–13^ other studies found no significant differences in the bone microarchitecture of people with T2D compared with those with a normal glucose metabolism (NGM).^14–16^ Still others even found favorable bone microarchitecture and strength parameters in T2D.^17^ Thus, previous studies using HRpQCT are inconsistent and unable to provide an unambiguous association between T2D and bone microarchitecture.

Therefore, we aimed to determine whether T2D and prediabetes are associated with impairments in HRpQCT parameters of bone microarchitecture and strength in the extensively phenotyped and relatively large cohort of The Maastricht Study. Based on the recent meta-analysis by Walle et al.,^9^ we hypothesize that T2D is associated with impairments in HRpQCT parameters. Additionally, we studied the association between measurements of glycemic control and the aforementioned HRpQCT parameters. Furthermore, we were able to include participants in the prediabetes state, a precursor of T2D, allowing us to study whether potential changes in T2D bone already manifest at an early stage, before T2D is diagnosed.

## Materials and methods

### Data source

We performed a cross-sectional analysis on data from the Maastricht study, an observational prospective population-based cohort study. The rationale and methodology have been described previously.^18^ In brief, the study focuses on the etiology, pathophysiology, complications, and comorbidities of T2D, and is characterized by an extensive phenotyping approach. All individuals aged between 40 and 75 yr and living in the southern part of the Netherlands were eligible. Participants were recruited through mass media campaigns, from the municipal registries and the regional Diabetes Patient Registry via mailings. Recruitment was stratified according to known T2D status, with an oversampling of individuals with T2D, for reasons of efficiency. The present report includes cross-sectional data from the first 7689 participants, who completed the baseline survey between November 2010 and December 2017. Examinations of each participant were performed within 3 mo after inclusion.

### Population

We included participants from the Maastricht Study with an HRpQCT scan at the distal radius and/or tibia. As the HRpQCT scan was added to the measurements of the Maastricht study in March 2015, participants who were included before this date were reinvited to the research center for an HRpQCT scan. The average lag-time (time between inclusion of the participant and the HRpQCT scan) was 1.78 (SD ± 2.35) yr. Clearance by the Dutch Ministry of Health was initially granted for HRpQCT scans of the distal radius and at a later stage for scans of the distal tibia. Therefore, 32.0% of the participants included in this study underwent an HRpQCT scan of the distal radius only (Supplementary Figure 1).

The study has been approved by the institutional medical ethical committee (NL31329.068.10) and the Minister of Health, Welfare and Sports of the Netherlands (Permit 131088-105234-PG). All participants gave written informed consent.

### Glucose metabolism status

To determine glucose metabolism status (GMS), all participants underwent a standardized 2-h 75-g oral glucose tolerance test (OGTT) after an overnight fast. For safety reasons, participants using insulin or with a fasting glucose level above 11.0 mmol/L, as determined by a finger prick, did not undergo the OGTT. For these individuals (*n* = 13), fasting glucose level and information about diabetes medication were used to determine GMS. GMS was defined according to the WHO 2006 criteria into NGM, impaired fasting glucose or impaired glucose tolerance, which were grouped together as prediabetes and T2D.^19^ Individuals without type 1 diabetes on glucose-lowering medication were classified as having T2D.^18^

Additionally, we studied several continuous measures of glycemic control. From the fasted blood draw, the HbA1c and FPG were determined for all participants. From the OGTT, we also determined plasma glucose 2-h postglucose load, the incremental glucose peak (IGP), and the Matsuda index. Some measures could only be determined in a subset of participants with multiple OGTT timepoints. The Matsuda index is a measure of insulin sensitivity. A higher value indicates greater insulin sensitivity, while a lower value suggests insulin resistance. Furthermore, skin autofluorescence (SAF) was measured and used as a proxy for AGE accumulation in a subset of the participants. Lastly, a subset of the participants was provided with a continuous glucose monitoring (CGM) device, from which the CGM assessed coefficient of variation (CGM-CV), a measure of the within-subject variability of blood glucose values, was derived.

### HRpQCT scans

The nondominant radius and ipsilateral tibia were scanned on an HRpQCT scanner (First generation, Xtreme-CT; Scanco Medical AG, Brüttisellen, Switzerland) using the standard in vivo protocol as described previously.^8^ If participants had previously sustained a fracture of the distal radius or tibia on the nondominant side, the dominant distal radius and/or tibia was scanned. According to the previously reported scan process,^20^ the reference line was placed on the radial joint surface and the endplate of the distal tibia, and all scans were graded regarding motion artifacts by a trained and experienced technician. For the analyses, only scans with a quality grade 1 to 3 (no, minor, or moderate motion artifacts) were used, while scans with quality grade 4 and 5 (severe or extreme motion artifacts) were excluded.^8^ Additionally, scans with an inadequate position of the reference line or with selection of the wrong scan protocol were excluded.

The scans were automatically contoured and segmented according to the procedure described previously.^20^ The following HRpQCT parameters were calculated from the images: cross-sectional area (CSA), total BMD (Tt.BMD), trabecular BMD (Tb.BMD), cortical BMD (Ct.BMD), trabecular number (Tb.N), trabecular thickness (Tb.Th), trabecular separation (Tb.Sp), and cortical thickness (Ct.Th). In addition, extended analysis of the cortical compartment was performed to obtain cortical pore volume (Ct.PoV), cortical porosity (Ct.Po), and cortical pore diameter (Ct.Po.Dm).^21^ Finally, microfinite element analysis was performed in Scanco Finite Element Software v1.15, as described previously.^22^^,^^23^ In short, all voxels representing bone tissue were converted into brick elements of the same size. A Young modulus of 10 GPa and a Poisson ratio of 0.3 were assigned to every element. Compression stiffness and estimated failure load were estimated by applying a virtual ‘high-friction’ compression test in the axial direction.^23^

### Standardized analyses

Standardized analyses allowed us to study the associations between GMS and measurements of glycemic control and HRpQCT parameters of bone microarchitecture and strength irrespective of units. The standardized scores (z-scores) of the bone compartment quality parameters were calculated by grouping the individuals into age categories (40-44, 45-49, 50-54, 55-59, 60-64, 65-69, 70-74, and >75 years old). Z-scores were calculated by subtracting the mean of the reference group (being the NGM group within that specific age category) from that individuals’ value and then dividing this by the SD of the reference group. These analyses were stratified by sex.

Measurements of glycemic control (HbA1c, FPG, plasma glucose 2-h postglucose load in the OGTT, IGP, SAF, CGM-CV, and the Matsuda index) were additionally standardized. To standardize these variables, z-scores were calculated in SPSS as follows: first subtracting the population mean from each individual value and then dividing each remainder by the population SD.

### Covariates

Age, body mass index (BMI), smoking status, alcohol use, educational level, use of medication that affects bone, estimated glomerular filtration rate (eGFR), moderate-to-vigorous physical activity (MVPA), history of cardiovascular disease, and lag-time were considered as potential confounders. Covariates were selected based on the influence their inclusion yielded on the beta (>10% change) or their known association with bone quality and T2D. BMI was calculated by dividing weight in kilogram by height in meters squared, which were measured while wearing light clothing without shoes, using a scale and stadiometer to the nearest 0.1 cm and 0.5 kg, respectively. Smoking status (never, former, current), alcohol use (none, low, high), cardiovascular disease and fracture history, and educational level (low, medium, high) were assessed using a questionnaire. The use of medication was assessed during interviews and MVPA was measured with an accelerometer. Lastly, eGFR was estimated with the CKD-EPI equation based on the combination of serum creatinine and serum cystatin C.

### Statistical analyses

General characteristics and HRpQCT bone compartment parameters were calculated for all three groups, being NGM, prediabetes, and T2D, separately. Data were additionally stratified by sex due to the inherent differences in bone metabolism and the uneven distribution of men and women per GMS group. Categorical variables are presented as number of participants with percent (*n*, %) and normally distributed continuous variables are presented as mean values with SD.

Interaction was evaluated and none were identified between GMS and BMI, and GMS and BMD measured by HRpQCT using a threshold of *p* <.10 for interaction.^24^

Linear regression analyses were employed to investigate the association between GMS and measurements of glycemic control, and bone compartment quality parameters, using both crude (age adjusted, model 1), partially adjusted (model 2), and fully adjusted (model 3) models. The normality assumption was checked, and if not met, data were log-transformed. All analyses were stratified by sex. The results from these analyses are all presented as beta’s, with 95% CIs.

The main analysis investigated the association between GMS and standardized bone compartment quality parameters, using the z-scores of the bone compartment quality parameters. For GMS, NGM was set as the reference group. The standardized betas obtained from this analysis represent the difference in HRpQCT outcome parameter in SDs for T2D or prediabetes versus NGM.

To control for multiple comparisons, we performed the false discovery rate (FDR) procedure as described by Benjamini-Hochberg et al.^25^ For this procedure, we assumed 30 comparisons were made: 15 parameters for the radius and 15 for the tibia in each group, being men and women with prediabetes or T2D.

Furthermore, we performed a linear regression analysis to investigate the association between measurements of glycemic control and the bone compartment quality parameters, where we used the standardized values of both, as explained above. Here, the standardized betas obtained from the analysis represent the difference in HRpQCT outcome parameter in SDs, per SD greater measure of glycemic control.

Additionally, we performed a linear regression analysis to study the association between diabetes duration and HbA1c, and bone compartment quality parameters within the T2D subgroup. Diabetes duration was assessed by a questionnaire where the onset of diabetes was recorded. The date of onset of diabetes was then used to calculate diabetes duration.

As a sensitivity analysis, we repeated the main analysis but only in the population with both a radius and tibia scan, so that both analyses were powered equally.

All analyses were performed with IBM Statistical Package for Social Sciences for Macintosh, version 25.0 (IBM SPSS, IBM Corp, Armonk, NY, USA). A two-sided *p*-value <.05 was considered statistically significant.

## Results

### Study population

A flowchart of the inclusion of participants in this study from the original cohort is presented in Supplementary Figure 1. In total, 1400 men (796 [56.9%] NGM, 228 [16.3%] prediabetes, and 376 [26.9%] T2D) and 1415 women (1014 [71.7%] NGM, 211 [14.9%] prediabetes, and 190 [13.4%] T2D) were included. The mean age was 59.8 ± 8.6 and 57.6 ± 9.0 yr for men and women, respectively (Table 1). The prevalence of a history of fractures was similar between individuals with an NGM (men: 41.8%; women: 33.1%) and T2D (men: 36.4%; women: 31.6%).

**Table 1:** General characteristics of the study population.

	**Men (*n* = 1400)**			**Women (*n* = 1415)**		
	**NGM (*n* = 796)**	**Prediabetes (*n* = 228)**	**T2D** **(*n* = 376)**	**NGM** **(*n* = 1014)**	**Prediabetes (*n* = 211)**	**T2D** **(*n* = 190)**
**Age (years)**	57.8 (8.8)	61.6 (8.1)	63.0 (7.2)	56.3 (8.6)	60.7 (9.1)	60.9 (8.3)
**BMI (kg/m** ^**2**^ **)**	26.0 (3.3)	27.9 (4.1)	29.3 (4.4)	25.4 (4.0)	27.6 (4.5)	30.2 (5.6)
**History of fracture**	333 (41.8%)	91 (39.9%)	137 (36.4%)	336 (33.1%)	67 (31.8%)	60 (31.6%)
**History of CVD**	87 (10.9%)	49 (21.5%)	119 (31.6%)	114 (11.2%)	29 (13.7%)	32 (16.8%)
**MVPA (h/week)**	6.0 (4.6)	5.1 (3.9)	4.2 (4.1)	6.1 (4.6)	4.8 (3.6)	4.9 (4.7)
**eGFR**	79.8 (13.3)	79.5 (14.3)	79.1 (16.5)	75.2 (12.8)	74.3 (14.1)	77.6 (18.4)
**aBMD LS (g/cm** ^**2**^ **)**	1.10 (0.19)	1.14 (0.20)	1.15 (0.19)	0.99 (0.15)	1.00 (0.16)	1.06 (0.20)
**aBMD TH (g/cm** ^**2**^ **)**	0.99 (0.13)	1.03 (0.13)	1.03 (0.17)	0.86 (0.12)	0.88 (0.14)	0.92 (0.14)
**aBMD FN (g/cm** ^**2**^ **)**	0.81 (0.13)	0.83 (0.13)	0.83 (0.18)	0.73 (0.11)	0.74 (0.12)	0.76 (0.13)
**Ethnicity**						
**Caucasian**	789 (99.1%)	227 (99.6%)	370 (98.4%)	1003 (98.9%)	209 (99.1%)	185 (97.4%)
**Other**	7 (0.9%)	1 (0.4%)	6 (1.6%)	11 (1.1%)	2 (0.9%)	5 (2.6%)
**Skin photo type**						
**Type I**	0 (0%)	0 (0%)	0 (0%)	0 (0%)	0 (0%)	0 (0%)
**Type II**	34 (4.3%)	14 (6.1%)	31 (8.2%)	53 (5.2%)	17 (8.1%)	22 (11.6%)
**Type III**	6668 (83.9%)	182 (79.8%)	290 (77.1%)	834 (82.2%)	167 (79.1%)	140 (73.7%)
**Type IV**	12 (1.5%)	7 (3.1%)	6 (1.6%)	25 (2.5%)	3 (1.4%)	4 (2.1%)
**Type V**	4 (0.5%)	0 (0%)	1 (0.3%)	4 (0.4%)	0 (0%)	0 (0%)
**Type VI**	3 (0.4%)	0 (0%)	1 (0.3%)	0 (0%)	0 (0%)	0 (0%)
**Missing**	75 (9.4%)	25 (11.0%)	47 (12.5%)	98 (9.7%)	24 (11.4%)	24 (12.6%)
**Educational level**						
**Low**	164 (20.6%)	62 (27.2%)	142 (37.8%)	314 (31.0%)	83 (39.3%)	97 (51.1%)
**Medium**	231 (29.0%)	63 (27.6%)	111 (29.5%)	319 (31.5%)	55 (26.1%)	53 (27.9%)
**High**	401 (50.4%)	103 (45.2%)	123 (32.7%)	381 (37.6%)	73 (34.6%)	40 (21.0%)
**Smoking status**						
**Never**	322 (40.5%)	69 (30.3%)	100 (26.6%)	446 (44.0%)	91 (43.1%)	81 (42.6%)
**Former**	361 (45.4%)	129 (56.6%)	227 (60.4%)	466 (46.0%)	104 (49.3%)	78 (41.1%)
**Current**	113 (14.2%)	30 (13.2%)	49 (13.0%)	102 (10.1%)	16 (7.6%)	31 (16.3%)
**Alcohol use**						
**None**	62 (7.8%)	25 (11.0%)	68 (18.1%)	220 (21.7%)	42 (19.9%)	75 (39.5%)
**Low**	562 (70.6%)	146 (64.0%)	230 (61.2%)	561 (55.3%)	114 (54.5%	84 (44.2%)
**High**	172 (21.6%)	57 (25.0%)	78 (20.7%)	233 (23.0%)	54 (25.6%)	31 (16.3%)
**Medication use**						
**Glucose-lowering drugs**	N/A	N/A	271 (72.1%)	N/A	N/A	113 (59.5%)
**Insulin**	N/A	N/A	43 (14.1%)	N/A	N/A	21 (11.1%)
**Oral glucose-lowering drugs**	N/A	N/A	259 (68.9%)	N/A	N/A	105 (55.3%)
**Biguanides**	N/A	N/A	249 (66.2%)	N/A	N/A	98 (51.6%)
**DPP4-inhibitors**	N/A	N/A	26 (6.9%)	N/A	N/A	7 (3.7%)
**GLP1-RAs**	N/A	N/A	4 (1.1%)	N/A	N/A	2 (1.1%)
**SGLT2-inhibitors**	N/A	N/A	1 (0.3%)	N/A	N/A	0 (0%)
**SUs**	N/A	N/A	75 (19.9%)	N/A	N/A	28 (14.7%)
**Thiazolidinediones**	N/A	N/A	2 (0.5%)	N/A	N/A	2 (1.1%)
**Others (excl. insulin)**	N/A	N/A	2 (0.5%)	N/A	N/A	2 (1.1%)
**Medication that affects bone**	44 (5.5%)	13 (5.7%)	38 (10.1%)	106 (10.5%)	19 (9.0%)	30 (15.8%)
**Anti-osteoporosis treatment**	9 (1.1%)	2 (0.9%)	4 (1.1%)	30 (3.0%)	11 (5.2%)	5 (2.6%)
**GMS characteristics**						
**HbA1c (mmol/mol)**	35.5 (3.8)	37.9 (4.4)	50.3 (9.8)	35.4 (3.8)	37.9 (4.5)	48.7 (9.5)
**HbA1c (%)**	5.4 (0.3)	5.6 (0.4)	6.8 (0.9)	5.4 (0.3)	5.6 (0.4)	6.6 (0.9)
**Diabetes duration at inclusion (years)**	N/A	N/A	6.3 (7.7)	N/A	N/A	4.2 (6.5)
**HRpQCT scans (n)**						
**Radius**	796 (100%)	228 (100%)	376 (100%)	1014 (100%)	211 (100%)	190 (100%)
**Tibia**	564 (70.9%)	152 (66.7%)	230 (61.2%)	731 (72.1%)	136 (64.5%)	102 (53.7%)
**Time gap between visit 1 and HR-pQCT scan (years)**	1.4 (2.1)	1.5 (2.2)	2.1 (2.5)	1.3 (2.0)	1.5 (2.2)	1.8 (2.4)

Data are presented as mean (SD) or number (%).

BMD, bone mineral density; BMI, body mass index; CVD, cardiovascular disease; eGFR, estimated glomerular filtration rate; HbA1c, glycated hemoglobin; HRpQCT, high resolution peripheral quantitative computed tomography; MVPA, moderate to vigorous physical activity; NGM, normal glucose metabolism; T2D, type 2 diabetes mellitus.

Medication that affects bone was defined as glucocorticoids, antidepressants, antipsychotics, neuroleptica.

### HRpQCT parameters (Z-scores) according to GMS

The descriptive statistics of the bone compartment quality parameters are presented in Table 2.

**Table 2:** HRpQCT parameters (mean ± SD) for men and women according to glucose metabolism status.

**Men, radius**			
**Grade 1- 3 scans**	**NGM (*n* = 796)**	**Prediabetes (*n* = 228)**	**T2D (*n* = 376)**
***Volumetric BMD***			
** Total BMD (mg HA/cm^3^)**	318.09 (56.19)	323.95 (54.66)	324.78 (58.24)
** Cortical BMD (mg HA/cm^3^)**	852.27 (59.81)	852.19 (56.05)	849.38 (55.49)
** Trabecular BMD (mg HA/cm^3^)**	187.04 (34.68)	188.08 (34.20)	188.62 (35.89)
***Areal parameters***			
** Total area (SE) (mm^2^)**	390.69 (60.66)	384.68 (58.51)	383.55 (63.07)
** Cortical area (SE) (mm^2^)**	68.38 (15.40)	70.62 (15.43)	70.45 (16.01)
** Trabecular area (SE) (mm^2^)**	316.29 (61.10)	308.30 (58.36)	307.36 (64.51)
***Microarchitecture***			
** Trabecular number (mm^−1^)**	2.03 (0.25)	2.08 (0.26)	2.10 (0.27)
** Trabecular thickness (mm)**	0.08 (0.01)	0.08 (0.01)	0.07 (0.01)
** Trabecular separation (mm)^†^**	0.41 (0.38; 0.46)	0.41 (0.37; 0.45)	0.39 (0.36; 0.44)
** Cortical thickness (SE) (mm)**	0.81 (0.20)	0.84 (0.19)	0.84 (0.21)
** Cortical pore volume (mm^3^)^†^**	16.89 (12.51; 23.17)	19.23 (14.33; 23.89)	19.42 (14.70; 24.67)
** Cortical porosity (%)^†^**	2.81 (2.09; 3.76)	3.01 (2.31; 3.90)	3.22 (2.44; 4.02)
** Cortical pore diameter (μm)^†^**	0.17 (0.16; 0.18)	0.17 (0.16; 0.18)	0.17 (0.16; 0.18)
***Strength***			
** Stiffness (N/mm)**	117.88 (21.87)	117.63 (21.39)	116.47 (20.50)
** Failure load (N)**	5616.66 (1009.87)	5601.80 (997.43)	5554.66 (950.15)
**Men, Tibia**			
**Grade 1- 3 scans**	**NGM (*n* = 564)**	**Prediabetes (*n* = 152)**	**T2D (*n* = 230)**
***Volumetric BMD***			
** Total BMD (mg HA/cm^3^)**	305.64 (51.30)	300.94 (47.33)	307.43 (48.66)
** Cortical BMD (mg HA/cm^3^)**	851.62 (47.09)	846.25 (51.36)	842.90 (47.88)
** Trabecular BMD (mg HA/cm^3^)**	190.75 (31.10)	189.03 (30.36)	190.94 (34.14)
***Areal parameters***			
** Total area (SE) (mm^2^)**	903.37 (138.42)	898.62 (132.62)	868.9309 (124.42)
** Cortical area (SE) (mm^2^)**	150.41 (30.83)	145.11 (29.66)	148.33 (30.76)
** Trabecular area (SE) (mm^2^)**	750.92 (142.40)	750.65 (137.10)	717.89 (128.17)
***Microarchitecture***			
** Trabecular number (mm^−1^)**	2.08 (0.29)	2.08 (0.29)	2.13 (0.31)
** Trabecular thickness (mm)**	0.08 (0.01)	0.08 (0.01)	0.08 (0.01)
** Trabecular separation (mm)^†^**	0.40 (0.36; 0.45)	0.40 (0.37; 0.45)	0.39 (0.36; 0.45)
** Cortical thickness (SE) (mm)**	1.30 (0.29)	1.26 (0.28)	1.31 (0.29)
** Cortical pore volume (mm^3^)^†^**	88.87 (70.88; 113.20)	87.68 (71.71; 107.66)	97.98 (78.51; 117.23)
** Cortical porosity (%)^†^**	7.25 (5.70; 8.84)	7.16 (5.67; 8.85)	7.77 (6.18; 9.44)
** Cortical pore diameter (μm)^†^**	0.18 (0.17; 0.20)	0.18 (0.17; 0.20)	0.18 (0.17; 0.19)
***Strength***			
** Stiffness (N/mm)**	287.99 (49.49)	281.92 (42.74)	278.24 (45.04)
** Failure load (N)**	13 666.35 (2260.63)	13 411.41 (1997.78)	13 232.13 (2064.76)
**Women, radius**			
**Grade 1- 3 scans**	**NGM (*n* = 1014)**	**Prediabetes (*n* = 210)**	**T2D (*n* = 189)**
***Volumetric BMD***			
** Total BMD (mg HA/cm^3^)**	288.67 (63.47)	294.75 (63.34)	306.20 (61.69)
** Cortical BMD (mg HA/cm^3^)**	858.93 (80.25)	848.55 (79.96)	859.18 (72.23)
** Trabecular BMD (mg HA/cm^3^)**	147.49 (36.00)	155.48 (37.07)	157.86 (33.49)
***Areal parameters***			
** Total area (SE) (mm^2^)**	274.16 (43.24)	269.38 (42.03)	274.16 (43.24)
** Cortical area (SE) (mm^2^)**	47.19 (11.43)	46.72 (11.69)	47.19 (11.43)
** Trabecular area (SE) (mm^2^)**	221.95 (44.21)	217.33 (42.11)	221.95 (44.21)
***Microarchitecture***			
** Trabecular number (mm^−1^)**	1.82 (0.31)	1.89 (0.32)	1.82 (0.31)
** Trabecular thickness (mm)**	0.07 (0.01)	0.07 (0.01)	0.07 (0.01)
** Trabecular separation (mm)^†^**	0.48 (0.42; 0.55)	0.45 (0.41; 0.52)	0.45 (0.40; 0.50)
** Cortical thickness (SE) (mm)**	0.68 (0.18)	0.68 (0.18)	0.68 (0.18)
** Cortical pore volume (mm^3^)^†^**	9.16 (6.55; 13.41)	10.91 (7.46; 14.45)	11.87 (8.63; 16.56)
** Cortical porosity (%)^†^**	2.14 (1.49; 3.27)	2.60 (1.66; 3.55)	2.71 (1.99; 3.84)
** Cortical pore diameter (μm)^†^**	0.17 (0.15; 0.18)	0.17 (0.16; 0.18)	0.17 (0.16; 0.19)
***Strength***			
** Stiffness (N/mm)**	75.38 (15.07)	75.37 (14.76)	75.38 (15.07)
** Failure load (N)**	3600.24 (701.57)	3601.43 (685.94)	3600.25 (701.57)
***Volumetric BMD***			
** Total BMD (mg HA/cm^3^)**	264.92 (51.11)	273.70 (53.64)	273.74 (47.78)
** Cortical BMD (mg HA/cm^3^)**	836.65 (72.58)	831.39 (66.73)	818.71 (74.46)
** Trabecular BMD (mg HA/cm^3^)**	158.82 (33.07)	165.50 (37.09)	164.59 (31.21)
***Areal parameters***			
** Total area (SE) (mm^2^)**	698.56 (106.43)	682.75 (110.91)	673.92 (104.49)
** Cortical area (SE) (mm^2^)**	100.65 (23.04)	102.10 (23.68)	100.49 (25.75)
** Trabecular area (SE) (mm^2^)**	593.70 (111.69)	576.13 (114.54)	567.27 (110.86)
***Microarchitecture***			
** Trabecular number (mm^−1^)**	1.82 (0.32)	1.87 (0.32)	1.86 (0.30)
** Trabecular thickness (mm)**	0.07 (0.01)	0.07 (0.01)	0.07 (0.01)
** Trabecular separation (mm)^†^**	0.48 (0.42; 0.56)	0.46 (0.40; 0.54)	0.46 (0.40; 0.54)
** Cortical thickness (SE) (mm)**	1.00 (0.26)	1.02 (0.26)	1.01 (0.29)
** Cortical pore volume (mm^3^)^†^**	60.72 (45.41; 75.58)	63.82 (46.74; 83.02)	66.33 (57.31; 83.64)
** Cortical porosity (%)^†^**	6.96 (4.81; 9.26)	7.15 (5.40; 9.55)	7.84 (5.97; 9.92)
** Cortical pore diameter (μm)^†^**	0.19 (0.18; 0.20)	0.18 (0.17; 0.20)	0.18 (0.19; 0.20)
***Strength***			
** Stiffness (N/mm)**	196.20 (33.29)	198.69 (38.46)	196.43 (33.01)
** Failure load (N)**	9358.80 (1533.55)	9467.28 (1769.73)	9358.83 (1554.76)

Data are shown as mean (SD) or median (IQR; IQR).

Abbreviations: NGM, normal glucose metabolism; SE, standard evaluation; T2D, type 2 diabetes; HA, hydroxyapatite; SE, standard evaluation.

^†^Variables were log transformed due to a nonnormal distribution.

### Radius

**Figure 1:**
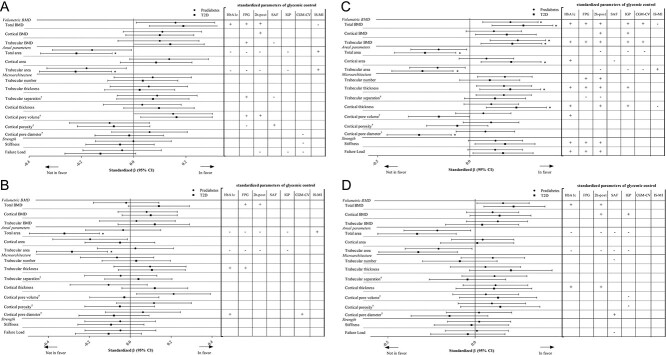
Linear regression analysis investigating the association between glucose metabolism status (GMS) and standardized HRpQCT parameters (z-scores), and the association between standardized parameters of glycemic control and standardized HRpQCT parameters. (A) Men, radius. (B) Men, tibia. (C) Women, radius. (D) Women, tibia. ^†^variables were log transformed due to a nonnormal distribution. Interpretation of the log transformed variables: Prediabetes/T2D is associated with an average change of 100×β% in the bone quality parameter. Additionally, these variables were inversed to conform to the “in favor” versus “not in favor” format. ^*^Variables are still significantly associated with GMS after controlling for multiple testing using the false discovery rate procedure. Analyses are adjusted for age, BMI, time gap between visit 1 and HRpQCT in months, educational level, use of medication that affects bone (glucocorticoids, antidepressants, antipsychotics, or neuroleptica), alcohol use, smoking status, use of anti-osteoporotic medication, eGFR, moderate-to-vigorous physical activity, and history of cardiovascular disease. 95% CI, 95% confidence interval; 2h-post, plasma glucose 2-h postglucose load; CGM-CV, continuous glucose monitoring assessed coefficient of variation; FPG, fasting plasma glucose, HbA1c, glycated hemoglobin; IGP, incremental glucose peak; IS-MI, insulin sensitivity measured as the Matsuda index; SAF, skin autofluorescence; T2D, type 2 diabetes. The standardized betas represent the difference in HRpQCT outcome parameter in SDs for T2D or prediabetes versus NGM.

After adjustment, T2D was associated with a statistically significant higher Tt.BMD (+0.15 SD in men and +0.31 SD in women) and Ct.Th (+0.16 SD in men and +0.25 SD in women) and a statistically significant smaller Tt.Ar (-0.22 SD in men and -0.24 SD in women) and Tb.Ar (-0.22 SD in men and -0.26 SD in women) compared with NGM at the distal radius in both men and women (Figure 1 and Supplementary Table 1).

In women, after adjustment, T2D was additionally associated with a statistically significant higher Ct.BMD (+0.19 SD), Tb.BMD (+0.27 SD), Ct.Ar (+0.22 SD), stiffness (+0.19 SD), failure load (+0.18 SD), Tb.Th (+0.22 SD), Ct.Po.V (+0.17 SD), and Ct.Po.Dm (+0.28 SD) compared with NGM.

The prediabetes state was, after adjustment, associated with a statistically significant higher Tt.BMD, but smaller Tt.Ar and Tb.Ar as compared with NGM, at the distal radius in both men and women. For women, prediabetes was additionally associated with a statistically significant higher Ct.BMD, Tb.N, and Tb.Th, and a significantly lower Tb.Sp compared with NGM.

After controlling for the multiple comparisons using the FDR procedure by Benjamini-Hochberg et al., T2D was associated with a statistically significant smaller Tt.Ar and Tb.Ar in men and women compared with NGM and after adjustment. In women, T2D was additionally associated with a statistically significant higher Tt.BMD, Tb.BMD, Ct.Ar, Tb.Th, Ct.Th, and Ct.Po.DM when compared with NGM and after adjustment. After adjustment, prediabetes was associated with a statistically significant higher Tt.BMD and Tb.BMD in women only compared with NGM.

### Tibia

After adjustments, T2D was associated with a significantly smaller Tt.Ar (-0.32 SD in men and -0.33 SD in women) and Tb.Ar (-0.29 in men and -0.33 SD in women) at the distal tibia, in both men and women, while only in women, T2D was additionally associated with a statistically significant higher Tt.BMD (+0.23 SD) compared with NGM.

In the distal tibia, the prediabetes state was associated with a statistically significant smaller Ct.Ar and Ct.Po.V in men compared with NGM and after adjustment. In women, prediabetes was associated with a significantly higher Ct.BMD but a statistically significant smaller Tt.Ar and Tb.Ar compared with NGM and after adjustment.

When taking into account the FDR for multiple comparisons, after adjustment, T2D was again associated with a smaller Tt.Ar and Tb.Ar in men but not women compared with NGM. For women, after adjustment, no significant associations were found after the FDR procedure. Prediabetes was also not significantly associated with any bone parameters after the FDR procedure and adjustments.

The results from the analyses with the untransformed HRpQCT variables (no Z-scores) are presented in Supplementary Table 2.

### Standardized measurements of glycemic control and HRpQCT parameters

In general, we found that a higher HbA1c, FPG, plasma glucose 2-h postglucose load in the OGTT and the IGP were associated with a statistically significant higher BMD measured by HRpQCT and Tb.Th, but a significantly lower Tt.Ar and Tb.Ar (Figure 1 and Supplementary Table 3). In women, we additionally observed that a higher HbA1c, FPG, and plasma glucose 2-h postglucose load in the OGTT were also associated with a statistically significant higher stiffness and failure load at the distal radius.

A higher CGM-CV was associated with a statistically significant higher BMD measured by HRpQCT in women at the distal radius, but significantly lower stiffness and failure load in men at the distal radius. Additionally, SAF showed inverse associations with bone area parameters, but significantly higher Ct.Po and Ct.PoV, although these associations were not consistently found across the four subgroups.

Lastly, the Matsuda index, in which a higher value indicates greater insulin sensitivity, was found to be statistically associated with lower BMD as measured by HRpQCT and a higher Tt.Ar and Tb.Ar.

### Diabetes duration and HbA1c, and bone quality within the T2D subgroup

At the distal radius, we did not find a significant association between T2D duration and HRpQCT parameters in men or women within the T2D cohort. In women, HbA1c was associated with a significantly higher failure load after adjustment (adjusted beta 12.74; 95% CI, 2.53 to 22.94). We found no significant association between HbA1c and any of the other HRpQCT parameters within the T2D subgroup.

### Sensitivity analysis

The sensitivity analysis including only individuals with both a radius and tibia scan showed similar results as to our main analysis.

## Discussion

We found that in this cohort of Maastricht study participants, bone microarchitecture and strength were not impaired in individuals with T2D compared with those without. The findings in this study were most pronounced in women, especially at the distal radius. After controlling for multiple comparisons using the FDR procedure, T2D was significantly associated with a smaller Tt.Ar and Tb.Ar in men and women compared with NGM. In women, T2D was additionally associated with a higher Tt.BMD, Tb.BMD, Ct.Ar, Tb.Th, Ct.Th, and Ct.Po.Dm when compared with NGM. Furthermore, women with prediabetes also had a higher Tt.BMD and Tb.BMD measured by HRpQCT. Furthermore, in women, we found parameters of glycemic control, for which higher values signify a worse glycemic control, to be positively associated with the density parameters, stiffness and failure load and negatively with Tb.Ar. Insulin sensitivity was negatively associated with Tt.BMD, Ct.BMD, and Ct.Th and positively associated with Tb.Ar, most pronounced at the distal radius in women.

Our study included the largest T2D cohort so far (376 T2D men and 190 T2D women) when compared with all previous studies, including the pooled data in the meta-analysis of Walle et al.^9^ Additionally, in contrast to most studies, we analyzed the HRpQCT outcomes of men and women separately. Given the sex differences, as found in our study, we believe that the data of men and women should be separated when studying bone microarchitecture.

Our results implicate that in this cohort of Maastricht study participants, T2D does not have a negative, and in women, possibly even a positive impact on bone microarchitecture but does lead to a smaller trabecular compartment. Our findings are partly in line with the recently published meta-analysis by Walle et al.,^9^ which included 516 T2D participants and 3067 controls. The results from this meta-analysis indicated a higher Tt.BMD and Tb.BMD, Tb.N, and Ct.Th in T2D, as in our study, but contrastingly reported a normal Tt.Ar and higher Ct.Po. Rasmussen et al.^17^ recently reported similar findings to our study, such as a higher Ct.Ar, and did not find an association with a higher Ct.Po in 96 participants with T2D. Our results are further supported by the study performed by Hunt et al.,^26^ who found that Young’s modulus, yield stress, and ultimate stress were higher in cancellous specimens from the femoral neck from T2D individuals (*n* = 11) compared with NGM individuals (*n* = 9).

One of our consistent findings was a smaller total and trabecular area, in men and women, at the radius and tibia. The Tt.Ar is made up of the Ct.Ar and the Tb.Ar, meaning that since we did not see a smaller Ct.Ar, the smaller Tt.Ar was fully explained by a smaller Tb.Ar. Although it has been postulated that the automated contours might misplace the endosteal contour, which separates the trabecular from the cortical bone,^27^ this does not detract from our finding of a decreased Tt.Ar.

In a recent meta-analysis, it was shown that of all the HRpQCT parameters, Ct.BMD, Tb.Th, and stiffness were the best predictors of fracture risk. Moreover, the non-weight-bearing distal radius was a more preferable site than distal tibia for fracture prediction.^28^ In our analyses, women with T2D showed a significantly higher Ct.BMD, Tb.Th, and stiffness at the distal radius compared with women without T2D, which aligns with our finding from a previous study where we found that risk of prevalent VFs was significantly lower in women with T2D, but not in men.^29^

Fracture risk was found to be increased in T2D in multiple studies,^3^^,^^30^^)^ for which several explanations have been brought forward. First, bone turnover has been reported to be decreased in T2D,^31^ which in turn is associated with osteoporosis and fracture risk.^5^ Indeed, Leanza et al.^32^ found that gene expression of sclerostin, a potent inhibitor of the canonical Wnt signaling pathway which regulates bone homeostasis, was upregulated in T2D. Moreover, both hyperinsulinemia in T2D and the previously mentioned higher sclerostin levels could stimulate the bone marrow adipogenesis, leading to an increase in bone marrow adipose tissue and senescence of bone marrow stromal stem cells.^6^^,^^33^ This, in turn, can result in lower bone quality.^6^^,^^34^ Unfortunately, data on bone turnover markers were not available in our cohort. Lastly, the long-term hyperglycemic state associated with T2D has been reported as an accelerant for the formation of AGEs.^35^ AGEs have been reported to have a number of negative effects on the bone and on bone turnover. First, AGEs can interfere with normal osteoblast function,^36^ impair osteoblast development,^37^ and inhibit the osteoclastic differentiation process.^38^ Moreover, AGEs can cause nonenzymatic cross-links of collagen type 1, decreasing the strength of the bone matrix.^7^^,^^39^ We looked at a possible association between AGEs and parameters of bone microarchitecture and strength by utilizing SAF as a proxy for AGE accumulation and did not find a significant association. However, brittleness of the bone due to the accumulation of nonenzymatic cross-links of collagen type 1 cannot be measured through HRpQCT scans, so the hypothesized decrease in tissue material properties via this pathway can neither be confirmed nor refuted based on the results from our study. SAF is a validated tool to assess AGE accumulation in skin;^40^ however, the measurement has some limitations. First, not all AGEs exhibit fluorescent properties. Second, sun exposure can be a source of skin photo-ageing and can be a confounding factor in AGE accumulation in the skin. Third, it is important to keep skin color in mind when working with SAF, as it can only be reliably measured in individuals with skin photo type I-IV.^41^ Of note, only four individuals with skin photo type VI were included in our study. Lastly, it is unknown how SAF translates to AGE accumulation in bone. Future studies are needed on the effect of AGEs, bone turnover, and bone marrow adipogenesis on bone material strength.

Our findings could be seen as somewhat counterintuitive to the previously mentioned older studies that reported an increased fracture risk in T2D.^3^^,^^42^ However, more recent literature reported different findings. The study by Axelsson et al.^43^ demonstrated that in the absence of certain risk factors such as a long diabetes duration or insulin treatment, individuals with T2D showed a lower fracture risk compared with the controls. Similarly, Wallander et al.^44^ found that only in individuals with T2D using insulin, fracture risk was significantly increased compared with individuals without T2D. In those with T2D on oral antidiabetics, fracture risk was found to be similar to the risk in individuals without T2D. Moreover, those with T2D not using any medication had a lower risk of hip fracture. Sarodnik et al.^45^ reported that the crude incidence of all fractures was marginally lower, but certainly not higher in patients with newly treated T2D compared with an age- and sex-matched control population. Lastly, in one of our previous study using the same Maastricht study cohort as in this study, we found that risk of prevalent VFs was significantly lower in women with T2D, but not in men.^29^ Thus, our findings concur with more recent literature on fracture risk in T2D.

When looking into the association between the Matsuda index, a measure expressing insulin sensitivity, and bone parameters, we found that a higher insulin sensitivity was associated with a lower BMD (as measured by HRpQCT). Similarly, Napoli et al.^46^ reported that insulin resistance was associated with a higher BMD but not consistently associated with an increased fracture risk. Furthermore, we found an association between insulin sensitivity and a larger area, which is in line with the study by Shanbhogue et al.^47^ This finding may indicate that a lower insulin sensitivity attributes to a smaller Tb.Ar, which would be consistent with our findings in individuals with prediabetes. Subjects with prediabetes generally have a lower insulin sensitivity but are not yet hyperglycemic. In our study, we found that prediabetes was associated with a smaller total and trabecular area, as was a lower insulin sensitivity.

In contrast to our study and the study of Rasmussen et al.,^17^ several studies have reported that Ct.Po was higher in T2D individuals.^11^^,^^12^^,^^48^^,^^49^ However, these studies included a limited number of individuals, ranging from 19 to 30 included individuals. It has been postulated that a higher Ct.Po in T2D would reflect the presence of diabetes-related complications, and the fact that the aforementioned studies included a limited number of participants may allow for a greater impact of diabetes-related complications.^9^ Considering our large study cohort, the results are likely more robust with a smaller impact of diabetes-related complications, especially since our cohort is considered to be relatively healthy.^50^ This could explain the absence of a significant association of T2D or the glycemic variables with Ct.Po. An alternative explanation could be differences in endosteal contour placement between studies,^51^ which could mean that trabeculae at the endosteal transition zone were misclassified as porous cortical bone in other studies.^52^ However, despite a possible misclassification of trabecular as cortical bone in our study, which could explain our finding of a smaller Tb.Ar, we did not find an increased Ct.Po.

Additionally, the findings in this study were more pronounced at the distal radius compared with the tibia, especially in women. This could be related to the fact that 32.0% of the included individuals underwent an HRpQCT scan at the distal radius only, which resulted in more power for the distal radius analyses. However, several studies reported lower strength parameters at the distal tibia, while others reported higher strength parameters at the radius, implying a nonweight bearing versus weight bearing effect.^17^^,^^52^ In our study, the observed associations could be attenuated in the tibia by a weight-bearing effect, since mechanical loading can increase an osteogenic bone response.^53^ Indeed, it has previously been shown that individuals with a fracture had a heterogeneous trabecular microarchitecture related to a loss of inner trabeculae, which was mitigated at the tibia compared with the radius.^54^ Moreover, our sensitivity analysis including only individuals with both a radius and tibia scan showed similar results as our main analysis, suggesting that the observed differences are not just due to differences in power.

When interpreting the results of our study, several limitations should be considered. First, this study has a cross-sectional design and the relationships between GMS and parameters of glycemic control and bone compartment quality parameters cannot demonstrate causality. Second, since the HRpQCT scan was added to the study design in a later phase, for some participants the baseline measurements were collected years before the HRpQCT scan was performed, meaning that some parameters of glycemic control might have changed at the time of HRpQCT scanning, although we did adjust for this lag-time. Furthermore, we did not look into the effect of specific classes of glucose-lowering drugs that could affect bone metabolism, such as thiazolidinediones (TZDs) and insulin. However, the use of TZDs in our cohort was very low (0.8%). Fourth, fat mass may influence HRpQCT density measurements due to beam-hardening effects caused by overlying fatty tissue,^55^^,^^56^ and fat mass is likely to be higher in our T2D participants. We corrected for BMI in our analyses, possibly negating some of this bias, but we cannot be certain that there was no lingering effect of fat mass on our measurements. Moreover, the HRpQCT scanner in our study was of the first generation, which is slightly inferior to the second-generation scanner in terms of resolution and amount of area scanned. Lastly, participants included in the Maastricht study are thought to be relatively healthy compared with the average T2D population due to a participation bias.^50^ Therefore, our results may not apply to a less well-controlled T2D population.

In conclusion, men and women with T2D showed a smaller total and trabecular area, and T2D was associated with a higher total BMD and trabecular BMD measured by HRpQCT, cortical area, trabecular thickness, cortical thickness, and cortical pore diameter in women compared with NGM. Women with prediabetes also showed a higher total BMD measured by HRpQCT and trabecular at the distal radius. HbA1c, FPG, and plasma glucose 2-h postglucose load in the OGTT were positively associated with total BMD measured by HRpQCT in men and women and with stiffness and failure load at the distal radius in women. Insulin sensitivity was negatively associated with BMD in women and positively associated with bone area in men and women.

## Supplementary Material

Appendix_HRpQCT_V2_ziae086

## Data Availability

The datasets presented in this article are not readily available because the dataset used in the present study was derived from the Maastricht Study. Upon reasonable request and with permission of the Maastricht Study management team, this dataset is available from the corresponding author. Requests to access the datasets should be directed to J.B., jvdbergh@viecuri.nl.
