# Supplementary material for: Bone microarchitecture and strength assessed by HRpQCT in individuals with type 2 diabetes and prediabetes: the Maastricht study
Source: JBMR Plus. 2024 Jul 3;8(9):ziae086. doi: 10.1093/jbmrpl/ziae086 (PMC11299510; doi:10.1093/jbmrpl/ziae086)
Supplement: Appendix_HRpQCT_V2_ziae086 [file appendix_hrpqct_v2_ziae086.docx]

Supplemental Figure 1. Flowchart illustrating the inclusion of participants included in the cohort


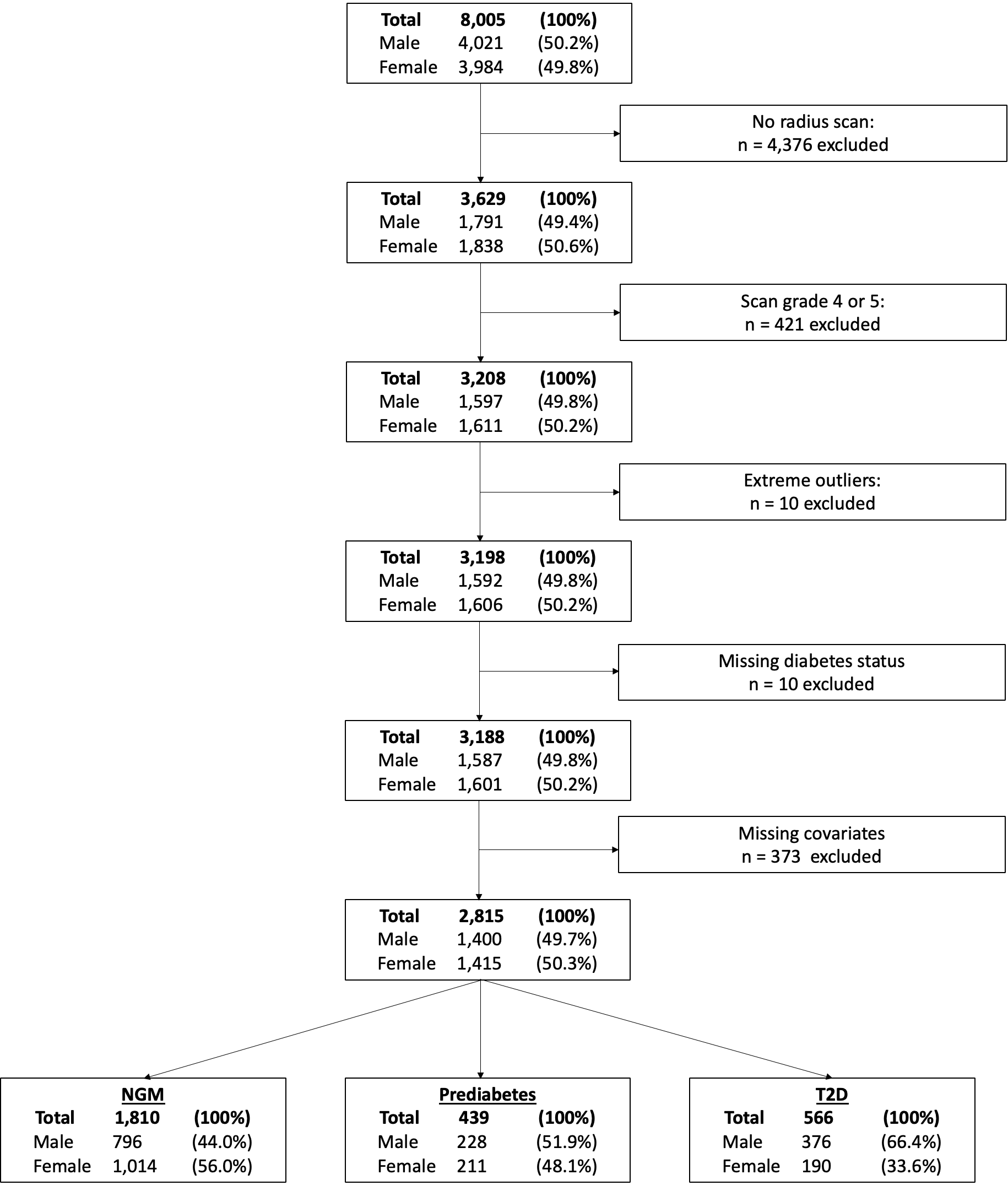


Supplemental Table 1. Linear regression analysis investigating the association between glucose metabolism status and standardized parameters of bone compartment quality (z-scores)

| **Men, radius** | | | | |
| --- | --- | --- | --- | --- |
| **Grade 1- 3 scans** | **Crude model** | | **Adjusted model*** | |
| *Volumetric BMD* | **Prediabetes (n=228)** | **T2D (n=376)** | **Prediabetes (n=228)** | **T2D (n=376)** |
| Total BMD (mg HA/cm^3^) | 0.19 (0.04; 0.34) | 0.24 (0.12; 0.37) | 0.16 (0.01; 0.31) | 0.19 (0.06; 0.33) |
| Cortical BMD (mg HA/cm^3^) | 0.12 (-0.02; 0.26) | 0.11 (-0.01; 0.23) | 0.11 (-0.04; 0.26) | 0.10 (-0.03; 0.23) |
| Trabecular BMD (mg HA/cm^3^) | 0.11 (-0.04; 0.26) | 0.16 (0.04; 0.29) | 0.10 (-0.06; 0.25) | 0.14 (0.00; 0.27) |
| *Areal parameters* |  |  |  |  |
| Total area (mm^2^) | -0.10 (-0.25; 0.05) | -0.12 (-0.25; 0.00) | -0.16 (-0.31; -0.01) | -0.22 (-0.36; -0.08) |
| Cortical area (mm^2^) | 0.22 (0.07; 0.37) | 0.25 (0.12; 0.37) | 0.14 (-0.02; 0.29) | 0.09 (-0.05; 0.22) |
| Trabecular area (mm^2^) | -0.15 (-0.30; 0.00) | -0.17 (-0.29; -0.04) | -0.18 (-0.33; -0.03) | -0.22 (-0.36; -0.08) |
| *Microarchitecture* |  |  |  |  |
| Trabecular number (mm^-1^) | 0.20 (0.05; 0.35) | 0.26 (0.14; 0.39) | 0.09 (-0.06; 0.24) | 0.07 (-0.07; 0.21) |
| Trabecular thickness (mm) | -0.03 (-0.17; 0.12) | -0.01 (-0.13; 0.12) | 0.03 (-0.12; 0.18) | 0.10 (-0.04; 0.23) |
| Trabecular separation (mm) ^†^ | 0.15 (0.01; 0.29) | 0.14 (0.02; 0.26) | 0.06 (-0.08; 0.21) | -0.02 (-0.15; 0.12) |
| Cortical thickness (mm) | 0.22 (0.07; 0.37) | 0.29 (0.16; 0.41) | 0.15 (0.00; 0.30) | 0.16 (0.03; 0.30) |
| Cortical pore volume (mm^3^) ^†^ | 0.04 (-0.10; 0.18) | 0.02 (-0.10; 0.14) | 0.00 (-0.15; 0.14) | -0.05 (-0.18; 0.08) |
| Cortical porosity (%)^†^ | -0.10 (-0.24; 0.05) | -0.21 (-0.33; -0.08) | -0.04 (-0.19; 0.11) | -0.13 (-0.26; 0.01) |
| Cortical pore diameter (μm) ^†^ | -0.19 (-0.34; -0.04) | -0.24 (-0.37; -0.12) | -0.09 (-0.25; 0.06) | -0.08 (-0.22; 0.06) |
| *Strength* |  |  |  |  |
| Stiffness (N/mm) | 0.13 (-0.02; 0.28) | 0.13 (0.01; 0.25) | 0.07 (-0.08; 0.22) | 0.04 (-0.10; 0.17) |
| Failure load (N) | 0.12 (-0.03; 0.27) | 0.13 (0.01; 0.25) | 0.06 (-0.09; 0.21) | 0.02 (-0.11; 0.16) |
| **Men, tibia** | | | | |
| **Grade 1- 3 scans** | **Crude model** | | **Adjusted model*** | |
| *Volumetric BMD* | **Prediabetes (n=152)** | **T2D (n=230)** | **Prediabetes (n=152)** | **T2D (n=230)** |
| Total BMD (mg HA/cm^3^) | -0.01 (-0.17; 0.15) | 0.15 (0.00; 0.29) | -0.02 (-0.18; 0.14) | 0.14 (-0.01; 0.29) |
| Cortical BMD (mg HA/cm^3^) | 0.09 (-0.06; 0.23) | 0.11 (-0.03; 0.24) | 0.08 (-0.06; 0.23) | 0.10 (-0.04; 0.23) |
| Trabecular BMD (mg HA/cm^3^) | 0.04 (-0.15; 0.22) | 0.11 (-0.06; 0.28) | 0.03 (-0.15; 0.22) | 0.12 (-0.06; 0.29) |
| *Areal parameters* |  |  |  |  |
| Total area (mm^2^) | 0.02 (-0.16; 0.21) | -0.21 (-0.37; -0.06) | -0.04 (-0.22; 0.15) | -0.32 (-0.49; -0.15) |
| Cortical area (mm^2^) | -0.10 (-0.28; 0.09) | 0.06 (-0.10; 0.21) | -0.20 (-0.38; -0.01) | -0.05 (-0.22; 0.12) |
| Trabecular area (mm^2^) | 0.06 (-0.12; 0.24) | -0.21 (-0.36; -0.05) | 0.03 (-0.16; 0.21) | -0.29 (-0.46; -0.11) |
| *Microarchitecture* |  |  |  |  |
| Trabecular number (mm^-1^) | 0.09 (-0.10; 0.28) | 0.29 (0.14; 0.45) | -0.07 (-0.24; 0.12) | 0.03 (-0.14; 0.20) |
| Trabecular thickness (mm) | -0.01 (-0.18; 0.17) | -0.06 (-0.21; 0.09) | 0.10 (-0.08; 0.27) | 0.11 (-0.06; 0.27) |
| Trabecular separation (mm) ^†^ | -0.16 (-0.34; 0.01) | 0.11 (-0.04; 0.26) | -0.21 (-0.39; -0.03) | 0.03 (-0.14; 0.20) |
| Cortical thickness (mm) | -0.11 (-0.29; 0.08) | 0.12 (-0.04; 0.28) | -0.11 (-0.29; 0.08) | 0.12 (-0.04; 0.28) |
| Cortical pore volume (mm^3^) ^†^ | -0.13 (-0.31; 0.05) | 0.06 (-0.09; 0.21) | -0.12 (-0.30; 0.07) | 0.05 (-0.12; 0.22) |
| Cortical porosity (%)^†^ | -0.22 (-0.41; -0.04) | -0.19 (-0.35; -0.03) | -0.06 (-0.25; 0.12) | 0.08 (-0.09; 0.25) |
| Cortical pore diameter (μm) ^†^ | -0.08 (-0.26; 0.10) | -0.26 (-0.42; -0.11) | 0.05 (-0.13; 0.22) | -0.04 (-0.20; 0.13) |
| *Strength* |  |  |  |  |
| Stiffness (N/mm) | 0.03 (-0.15; 0.20) | 0.00 (-0.15; 0.15) | -0.05 (-0.22; 0.12) | -0.09 (-0.26; 0.07) |
| Failure load (N) | 0.04 (-0.14; 0.22) | 0.01 (-0.15; 0.16) | -0.05 (-0.22; 0.13) | -0.11 (-0.24; 0.12) |
| **Women, radius** | | | | |
| **Grade 1- 3 scans** | **Crude model** | | **Adjusted model*** | |
| *Volumetric BMD* | **Prediabetes (n=211)** | **T2D (n=190)** | **Prediabetes (n=211)** | **T2D (n=190)** |
| Total BMD (mg HA/cm^3^) | 0.31 (0.16; 0.46) | 0.51 (0.36; 0.67) | 0.22 (0.07; 0.37) | 0.29 (0.12; 0.45) |
| Cortical BMD (mg HA/cm^3^) | 0.20 (0.06; 0.35) | 0.36 (0.21; 0.52) | 0.13 (-0.02; 0.28) | 0.20 (0.03; 0.36) |
| Trabecular BMD (mg HA/cm^3^) | 0.32 (0.17; 0.47) | 0.38 (0.23; 0.54) | 0.26 (0.11; 0.41) | 0.23 (0.06; 0.39) |
| *Areal parameters* |  |  |  |  |
| Total area (mm^2^) | -0.16 (-0.31; -0.01) | -0.24 (-0.39; -0.08) | -0.17 (-0.32; -0.02) | -0.24 (-0.41; -0.08) |
| Cortical area (mm^2^) | 0.23 (0.07; 0.38) | 0.54 (0.38; 0.69) | 0.09 (-0.06; 0.24) | 0.22 (0.06; 0.39) |
| Trabecular area (mm^2^) | -0.19 (-0.34; -0.04) | -0.31 (-0.46; -0.15) | -0.18 (-0.33; 0.03) | -0.26 (-0.42; -0.09) |
| *Microarchitecture* |  |  |  |  |
| Trabecular number (mm^-1^) | 0.30 (0.15; 0.45) | 0.35 (0.20; 0.51) | 0.19 (0.04; 0.34) | 0.11 (-0.06; 0.27) |
| Trabecular thickness (mm) | 0.17 (0.03; 0.32) | 0.20 (0.05; 0.35) | 0.19 (0.04; 0.34) | 0.22 (0.05; 0.38) |
| Trabecular separation (mm) ^†^ | -0.03 (-0.18; 0.12) | 0.28 (0.12; 0.43) | -0.07 (-0.22; 0.08 | 0.17 (0.00; 0.33) |
| Cortical thickness (mm) | 0.24 (0.09; 0.39) | 0.54 (0.38; 0.70) | 0.12 (-0.03; 0.26) | 0.25 (0.09; 0.42) |
| Cortical pore volume (mm^3^) ^†^ | -0.13 (-0.28; 0.02) | 0.04 (-0.12; 0.19) | -0.12 (-0.27; 0.04) | 0.07 (-0.10; 0.24) |
| Cortical porosity (%)^†^ | -0.11 (-0.26; 0.05) | 0.25 (0.9; 0.41) | -0.08 (-0.24; 0.07) | 0.28 (0.10; 0.45) |
| Cortical pore diameter (μm) ^†^ | -0.28 (-0.44; -0.13) | -0.36 (-0.52; -0.20) | -0.18 (-0.33; 0.03) | -0.14 (-0.30; 0.03) |
| *Strength* |  |  |  |  |
| Stiffness (N/mm) | 0.24 (0.09; 0.39) | 0.41 (0.25; 0.56) | 0.14 (-0.01; 0.29) | 0.19 (0.03; 0.35) |
| Failure load (N) | 0.24 (0.09; 0.39) | 0.40 (0.24; 0.56) | 0.14 (-0.01; 0.29) | 0.18 (0.02; 0.34) |
| **Women, tibia** | | | | |
| **Grade 1- 3 scans** | **Crude model** | | **Adjusted model*** | |
| *Volumetric BMD* | **Prediabetes (n=136)** | **T2D (n=102)** | **Prediabetes (n=136)** | **T2D (n=102)** |
| Total BMD (mg HA/cm^3^) | 0.30 (0.15; 0.44) | 0.42 (0.25; 0.59) | 0.16 (0.01; 0.31) | 0.22 (0.05; 0.40) |
| Cortical BMD (mg HA/cm^3^) | 0.18 (0.04; 0.33) | 0.28 (0.11; 0.45) | 0.10 (-0.05; 0.25) | 0.14 (-0.03; 0.32) |
| Trabecular BMD (mg HA/cm^3^) | 0.24 (0.07; 0.42) | 0.20 (0.00; 0.40) | 0.13 (-0.05; 0.30) | 0.04 (-0.17; 0.25) |
| *Areal parameters* |  |  |  |  |
| Total area (mm^2^) | -0.19 (-0.37; 0.00) | -0.34 (-0.55; -0.13) | -0.22 (-0.41; -0.03) | -0.33 (-0.56; -0.11) |
| Cortical area (mm^2^) | 0.28 (0.09; 0.47) | 0.38 (0.17; 0.59) | 0.04 (-0.14; 0.22) | 0.01 (-0.20; 0.22) |
| Trabecular area (mm^2^) | -0.22 (-0.41; -0.03) | -0.39 (-0.60; -0.18) | -0.21 (-0.40; - 0.02) | -0.33 (-0.55; -0.10) |
| *Microarchitecture* |  |  |  |  |
| Trabecular number (mm^-1^) | 0.26 (0.07; 0.44) | 0.18 (-0.03; 0.39) | 0.10 (-0.08; 0.28) | -0.09 (-0.30; 0.13) |
| Trabecular thickness (mm) | 0.05 (-0.14; 0.25) | 0.15 (-0.07; 0.37) | 0.06 (-0.14; 0.26) | 0.21 (-0.03; 0.44) |
| Trabecular separation (mm) ^†^ | 0.03 (-0.15; 0.22) | 0.03 (-0.18; 0.24) | -0.06 (-0.25; 0.13) | -0.13 (-0.35; 0.09) |
| Cortical thickness (mm) | 0.31 (0.12; 0.50) | 0.43 (0.22; 0.64) | 0.12 (-0.07; 0.30) | 0.11 (-0.11; 0.33) |
| Cortical pore volume (mm^3^) ^†^ | -0.14 (-0.33; 0.05) | -0.20 (-0.41; 0.01) | -0.10 (-0.29; 0.10) | -0.14 (-0.37; 0.08) |
| Cortical porosity (%)^†^ | -0.12 (-0.30; 0.07) | -0.03 (-0.24; 0.18) | -0.02 (-0.21; 0.17) | 0.15 (-0.07; 0.37) |
| Cortical pore diameter (μm) ^†^ | -0.26 (-0.45; -0.07) | -0.21 (-0.42; 0.01) | -0.11 (-0.30; 0.08) | 0.04 (-0.18; 0.26) |
| *Strength* |  |  |  |  |
| Stiffness (N/mm) | 0.21 (0.02; 0.40) | 0.22 (0.01; 0.43) | 0.02 (-0.16; 0.20) | -0.02 (-0.23; 0.19) |
| Failure load (N) | 0.21 (0.02; 0.40) | 0.21 (0.00; 0.42) | 0.01 (-0.17; 0.19) | -0.04 (-0.26; 0.17) |

Data are shown as beta’s, with 95% CIs.

^†^ Interpretation of the log transformed variables: prediabetes / T2D is associated with an average change of 100×β% in the bone quality parameter.

* Adjusted for age, BMI, time gap between visit 1 and HR-pQCT in months, educational level, use of medication that affects bone (glucocorticoids, antidepressants, antipsychotics or neuroleptica), alcohol use, smoking status, use of anti-osteoporotic medication, eGFR, moderate-to-vigorous physical activity, and history of cardiovascular disease.

BMD, bone mineral density; HA, hydroxyapatite; T2D, type 2 diabetes.

Supplemental Table 2. Linear regression analysis investigating the association between glucose metabolism status and bone compartment quality parameters

| **Men, radius** | | | | | | |
| --- | --- | --- | --- | --- | --- | --- |
| **Grade 1- 3 scans** | **Age adjusted model (Model 1)** | | **Model 2 *** | | **Model 3 **** | |
| *Volumetric BMD* | **Prediabetes (n=228)** | **T2D (n=376)** | **Prediabetes (n=228)** | **T2D (n=376)** | **Prediabetes (n=228)** | **T2D (n=376)** |
| Total BMD (mg HA/cm^3^) | 10.10 (1.77; 18.43) ^a^ | 12.51 (5.38; 19.64) ^a^ | 10.32 (2.01; 18.63) ^a^ | 13.09 (5.90; 20.29) ^a^ | 8.78 (0.30; 17.25) ^a^ | 10.17 (2.38; 17.96) ^a^ |
| Cortical BMD (mg HA/cm^3^) | 6.90 (-1.48; 15.28) | 6.96 (-0.21; 14.13) | 7.00 (-1.36; 5.37) | 7.39 (0.15; 14.63) | 6.59 (-1.97; 15.15) | 6.44 (-1.43; 14.31) |
| Trabecular BMD (mg HA/cm^3^) | 3.59 (-1.55; 8.73) | 5.09 (0.69; 9.49) | 3.78 (-1.35; 8.91) | 5.64 (1.20; 10.08) ^a^ | 3.08 (-2.14; 8.29) | 4.33 (-0.47; 9.12) |
| *Areal parameters* |  |  |  |  |  |  |
| Total area (mm^2^) | -6.46 (-15.55; 2.63) | -7.38 (-15.16; 0.40) | -6.93 (-15.99; 2.13) | -8.84 (-16.69; -1.00) ^a^ | -10.42 (-19.63; -1.20) ^a^ | -14.23 (-22.71; -5.76) ^a^ |
| Cortical area (mm^2^) | 3.13 (0.82; 5.43) ^a^ | 3.39 (1.42; 5.36) ^a^ | 3.07 (0.77; 5.37) ^a^ | 3.19 (1.20; 5.18) ^a^ | 1.76 (-0.56; 4.08) | 0.98 (-1.16; 3.11) |
| Trabecular area (mm^2^) | -8.83 (-18.02; 0.36) | -9.80 (-17.66; -1.93) ^a^ | -9.23 (-18.39; -0.07) ^a^ | -11.00 (-18.93; -3.07) ^a^ | -11.48 (-20.83; -2.13) ^a^ | -14.31 (-22.91; -5.71) ^a^ |
| *Microarchitecture* |  |  |  |  |  |  |
| Trabecular number (mm^-1^) | 0.06 (0.02; 0.09) ^a^ | 0.07 (0.04; 0.11) ^a^ | 0.05 (0.02; 0.09) ^a^ | 0.07 (0.04; 0.10) ^a^ | 0.03 (-0.01; 0.07) | 0.03 (-0.01; 0.06) |
| Trabecular thickness (mm) | 0.00 (0.00; 0.00) | 0.00 (0.00; 0.00) | 0.00 (0.00; 0.00) | 0.00 (0.00; 0.00) | 0.00 (0.00; 0.00) | 0.00 (0.00; 0.00) |
| Trabecular separation (mm) ^†^ | -0.03 (-0.05; -0.01) ^a^ | -0.04 (-0.06; -0.02) ^a^ | -0.03 (-0.05; -0.01) ^a^ | -0.04 (-0.06; -0.02) ^a^ | -0.02 (-0.04; 0.01) | -0.02 (-0.04; 0.01) |
| Cortical thickness (mm) | 0.04 (0.01; 0.07) ^a^ | 0.05 (0.03; 0.08) ^a^ | 0.04 (0.01; 0.07) ^a^ | 0.05 (0.03; 0.08) ^a^ | 0.03 (0.00; 0.06) | 0.03 (0.00; 0.06) |
| Cortical pore volume (mm^3^) ^†^ | 0.05 (-0.01; 0.12) | 0.05 (0.00; 0.11) | 0.05 (-0.01; 0.11) | 0.05 (-0.01; 0.10) | 0.01 (-0.05; 0.08) | -0.02 (-0.08; 0.04) |
| Cortical porosity (%)^†^ | 0.01 (-0.04; 0.07) | 0.01 (-0.04; 0.06) | 0.01 (-0.05; 0.07) | 0.01 (-0.04; 0.05) | -0.01 (-0.06; 0.05) | -0.03 (-0.08; 0.03) |
| Cortical pore diameter (μm) ^†^ | -0.01 (-0.03; 0.00) | -0.02 (-0.04; -0.01) ^a^ | -0.01 (-0.03; 0.00) | -0.02 (-0.04; -0.01) ^a^ | -0.01 (-0.02; 0.01) | -0.02 (-0.03; 0.00) |
| *Strength* |  |  |  |  |  |  |
| Stiffness (N/mm) | 1.99 (-1.12; 5.09) | 1.97 (-0.69; 4.63) | 1.97 (-1.13; 5.07) | 1.91 (-0.78; 4.59) | 0.78 (-2.36; 3.92) | -0.12 (-3.01; 2.77) |
| Failure load (N) | 88.66 (-55.26; 232.57) | 94.67 (-28.53; 217.86) | 86.87 (-56.72; 230.46) | 88.82 (-35.52; 213.15) | 26.51 (-118.53; 171.56) | -13.07 (-146.45; 120.30) |
| **Men, tibia** | | | | | | |
| **Grade 1- 3 scans** | **Age adjusted model (Model 1)** | | **Model 2 *** | | **Model 3 **** | |
| *Volumetric BMD* | **Prediabetes (n=152)** | **T2D (n=230)** | **Prediabetes (n=152)** | **T2D (n=230)** | **Prediabetes (n=152)** | **T2D (n=230)** |
| Total BMD (mg HA/cm^3^) | 1.03 (-7.92; 9.98) | 9.41 (1.32; 17.50) ^a^ | 0.44 (-8.45; 9.32) | 9.25 (1.17; 17.33) ^a^ | -0.57 (-9.59; 8.45) | 7.88 (-0.57; 16.33) |
| Cortical BMD (mg HA/cm^3^) | 3.26 (-4.98; 11.51) | 5.78 (-1.68; 13.24) | 2.91 (-5.35; 11.16) | 5.01 (-2.50; 12.52) | 2.63 (-5.81; 11.08) | 4.49 (-3.42; 12.40) |
| Trabecular BMD (mg HA/cm^3^) | 1.11 (-5.16; 7.38) | 2.65 (-3.03; 8.32) | 0.89 (-5.30; 7.08) | 3.33 (-2.30; 8.96) | 0.68 (-5.62; 6.98) | 3.18 (-2.72; 9.09) |
| *Areal parameters* |  |  |  |  |  |  |
| Total area (mm^2^) | -4.21 (-28.72; 20.30) | -38.36 (-60.53; -16.19) ^a^ | -2.90 (-27.42; 21.62) | -35.56 (-57.87; -13.25) ^a^ | -13.28 (-38.04; 11.48) | -46.96 (-70.15; -23.76) ^a^ |
| Cortical area (mm^2^) | -1.77 (-7.25; 3.70) | 2.92 (-2.03; 7.87) | -2.10 (-7.55; 3.36) | 2.52 (-2.44; 7.49) | -4.83 (-10.27; 0.61) | -0.81 (-5.91; 4.29) |
| Trabecular area (mm^2^) | -2.33 (-27.55; 22.90) | -40.49 (-63.30; -17.67) ^a^ | -0.75 (-25.96; 24.47) | -37.30 (-60.24; -14.36) ^a^ | -8.27 (-33.90; 17.36) | -45.20 (-69.21; -21.19) ^a^ |
| *Microarchitecture* |  |  |  |  |  |  |
| Trabecular number (mm^-1^) | 0.02 (-0.03; 0.07) | 0.05 (0.01; 0.10) ^a^ | 0.02 (-0.03; 0.07) | 0.06 (0.01; 0.10) ^a^ | -0.02 (-0.07; 0.03) | 0.01 (-0.04; 0.06) |
| Trabecular thickness (mm) | 0.00 (0.00; 0.00) | 0.00 (0.00; 0.00) | 0.00 (0.00; 0.00) | 0.00 (0.00; 0.00) | 0.00 (0.00; 0.00) | 0.00 (0.00; 0.00) |
| Trabecular separation (mm) ^†^ | -0.01 (-0.04; 0.02) | -0.03 (-0.05; 0.00) | -0.01 (-0.04; 0.02) | -0.03 (-0.06; 0.00) | 0.01 (-0.02; 0.04) | -0.01 (-0.03; 0.02) |
| Cortical thickness (mm) | -0.01 (-0.06; 0.04) | 0.05 (0.01; 0.10) ^a^ | -0.02 (-0.07; 0.04) | 0.04 (0.00; 0.10) | -0.03 (-0.08; 0.02) | 0.03 (-0.02; 0.08) |
| Cortical pore volume (mm^3^) ^†^ | -0.06 (-0.12; 0.00) | 0.02 (-0.04; 0.07) | -0.06 (-0.12; 0.00) | 0.02 (-0.04; 0.08) | -0.08 (-0.14; -0.02) ^a^ | 0.00 (-0.06; 0.06) |
| Cortical porosity (%)^†^ | -0.05 (-0.11; 0.00) | -0.01 (-0.06; 0.04) | -0.05 (-0.11; 0.01) | 0.00 (-0.05; 0.05) | -0.05 (-0.10; 0.01) | 0.01 (-0.05; 0.06) |
| Cortical pore diameter (μm) ^†^ | -0.02 (-0.04; 0.00) | -0.01 (-0.03; 0.00) | -0.02 (-0.04; 0.00) | -0.01 (-0.03; 0.00) | -0.01 (-0.02; 0.01) | 0.01 (-0.01; 0.02) |
| *Strength* |  |  |  |  |  |  |
| Stiffness (N/mm) | -0.11 (-8.56; 8.34) | -2.33 (-9.97; 5.32) | -0.36 (-8.73; 8.01) | -1.60 (-9.22; 6.01) | -3.54 (-11.93; 4.85) | -5.10 (-12.96; 2.76) |
| Failure load (N) | 22.95 (-364.05; 409.95) | -95.05 (-445.05; 254.96) | 13.10 (-370.50; 396.69) | -61.46 (-410.50; 287.59) | -160.90 (543.79; 221.99) | -257.90 (-616.64; 100.84) |
| **Women, radius** | | | | | | |
| **Grade 1- 3 scans** | **Age adjusted model (Model 1)** | | **Model 2 *** | | **Model 3 **** | |
| *Volumetric BMD* | **Prediabetes (n=211)** | **T2D (n=190)** | **Prediabetes (n=211)** | **T2D (n=190)** | **Prediabetes (n=211)** | **T2D (n=190)** |
| Total BMD (mg HA/cm^3^) | 17.77 (8.85; 26.69) ^a^ | 30.18 (20.84; 39.52) ^a^ | 12.34 (2.32 - 22.37) ^a^ | 24.49 (13.99 - 34.99) ^a^ | 11.41 (6.11 - 16.71) ^a^ | 14.19 (8.64 - 19.75) ^a^ |
| Cortical BMD (mg HA/cm^3^) | 17.60 (8.69; 26.52) ^a^ | 29.32 (19.88; 38.75) ^a^ | 12.01 (2.02 - 22.00) ^a^ | 22.87 (12.29 - 33.44) ^a^ | 11.44 (6.13 - 16.75) ^a^ | 14.72 (9.10 - 20.34) ^a^ |
| Trabecular BMD (mg HA/cm^3^) | 12.30 (3.42; 21.18) ^a^ | 16.69 (6.87; 26.50) ^a^ | 8.11 (-1.99 - 18.22) | 13.79 (2.61 - 24.96) ^a^ | 8.99 (3.70 - 14.27) ^a^ | 8.58 (2.73 - 14.42) ^a^ |
| *Areal parameters* |  |  |  |  |  |  |
| Total area (mm^2^) | -6.05 (-12.61; 0.50) | -8.95 (-15.83; -2.07) ^a^ | -5.91 (-12.64; 0.64) | -8.20 (-15.15; -1.26) ^a^ | -6.45 (-13.08; 0.17) | -9.49 (-16.81; -2.16) ^a^ |
| Cortical area (mm^2^) | 1.97 (0.39; 3.55) ^a^ | 4.69 (3.04; 6.35) ^a^ | 1.94 (0.36; 3.52) ^a^ | 4.44 (2.76; 6.11) ^a^ | 0.71 (-0.83; 2.26) | 1.64 (-0.06; 3.35) |
| Trabecular area (mm^2^) | -7.41 (-14.03; -0.78) ^a^ | -12.12 (-19.06; -5.18) ^a^ | -7.26 (-13.88; -0.64) ^a^ | -11.18 (-18.18; -4.17) ^a^ | -6.80 (-13.52; -0.08) ^a^ | -10.23 (-17.65; -2.80) ^a^ |
| *Microarchitecture* |  |  |  |  |  |  |
| Trabecular number (mm^-1^) | 0.09 (0.05; 0.14) ^a^ | 0.12 (0.07; 0.17) ^a^ | 0.09 (0.05; 0.14) ^a^ | 0.12 (0.07; 0.17) ^a^ | 0.06 (0.01; 0.10) ^a^ | 0.04 (-0.01; 0.09) |
| Trabecular thickness (mm) | 0.00 (0.00; 0.00) | 0.00 (0.00; 0.00) | 0.00 (0.00; 0.00) | 0.00 (0.00; 0.00) | 0.00 (0.00; 0.00) | 0.00 (0.00; 0.00) |
| Trabecular separation (mm) ^†^ | -0.06 (-0.09; -0.03) ^a^ | -0.08 (-0.12; -0.05) ^a^ | -0.06 (-0.09; -0.03) ^a^ | -0.08 (-0.12; -0.05) ^a^ | -0.04 (-0.07; -0.01) ^a^ | -0.03 (-0.07; 0.01) |
| Cortical thickness (mm) | 0.03 (0.01; 0.06) ^a^ | 0.08 (0.05; 0.10) ^a^ | 0.03 (0.01; 0.06) ^a^ | 0.07 (0.05; 0.10) ^a^ | 0.02 (-0.01; 0.04) | 0.03 (0.01; 0.06) ^a^ |
| Cortical pore volume (mm^3^) ^†^ | -0.03 (-0.10; 0.04) | 0.09 (0.02; 0.16) ^a^ | -0.03 (-0.10; 0.04) | 0.08 (0.01; 0.15) ^a^ | -0.05 (-0.11; 0.02) | 0.04 (-0.03; 0.12) |
| Cortical porosity (%)^†^ | -0.07 (-0.14; 0.00) | 0.00 (-0.07; 0.07) | -0.07 (-0.13; 0.00) | 0.00 (-0.07; 0.07) | -0.06 (-0.13; 0.01) | 0.01 (-0.06; 0.09) |
| Cortical pore diameter (μm) ^†^ | -0.01 (-0.03; 0.01) | 0.02 (0.01; 0.04) ^a^ | -0.01 (-0.03; 0.01) | 0.02 (0.00; 0.04) | -0.01 (-0.03; 0.01) | 0.03 (0.01; 0.05) ^a^ |
| *Strength* |  |  |  |  |  |  |
| Stiffness (N/mm) | 3.07 (0.99; 5.16) ^a^ | 5.49 (3.31; 7.68) ^a^ | 3.07 (0.99; 5.16) ^a^ | 5.63 (3.43; 7.84) ^a^ | 1.77 (-0.26; 3.81) | 2.59 (0.35; 4.84) ^a^ |
| Failure load (N) | 144.40 (47.34; 241.47) ^a^ | 253.81 (152.13; 355.48) ^a^ | 144.47 (47.33; 241.62) ^a^ | 260.29 (157.45; 363.12) ^a^ | 82.04 (-12.45; 176.53) | 116.62 (12.19; 221.05) ^a^ |
| **Women, tibia** | | | | | | |
| **Grade 1- 3 scans** | **Age adjusted model (Model 1)** | | **Model 2 *** | | **Model 3 **** | |
| *Volumetric BMD* | **Prediabetes (n=136)** | **T2D (n=102)** | **Prediabetes (n=136)** | **T2D (n=102)** | **Prediabetes (n=136)** | **T2D (n=102)** |
| Total BMD (mg HA/cm^3^) | 16.38 (7.64; 25.12) ^a^ | 22.29 (12.22; 32.37) ^a^ | 3.08 (3.18; 22.97) ^a^ | 15.56 (4.16; 26.97) ^a^ | 8.12 (1.92; 14.32) ^a^ | 8.94 (1.80; 16.08) ^a^ |
| Cortical BMD (mg HA/cm^3^) | 16.25 (7.51; 24.99) ^a^ | 21.25 (11.03; 31.46) ^a^ | 12.54 (2.70; 22.38) ^a^ | 12.75 (1.25; 24.25) ^a^ | 8.18 (1.97; 14.39) ^a^ | 9.18 (1.92; 16.43) ^a^ |
| Trabecular BMD (mg HA/cm^3^) | 9.14 (0.42; 17.85) ^a^ | 11.46 (1.13; 21.79) ^a^ | 8.01 (2.01; 18.04) ^a^ | 7.49 (4.39; 19.37) ^a^ | 4.425 (-1.81; 10.66) | 3.80 (-3.58; 11.19) |
| *Areal parameters* |  |  |  |  |  |  |
| Total area (mm^2^) | -19.20 (-38.90; 0.51) | -28.87 (-51.58; -6.16) ^a^ | -18.70 (-38.43; 1.02) | -25.53 (-48.59; -2.48) ^a^ | -23.26 (-43.43; -3.09) ^a^ | -31.32 (-55.22; -7.43) ^a^ |
| Cortical area (mm^2^) | 5.82 (2.03; 9.61) ^a^ | 6.89 (2.52; 11.26) ^a^ | 5.81 (2.02; 9.59) ^a^ | 6.61 (2.19; 11.03) ^a^ | 1.34 (-2.27; 4.96) | 0.74 (-3.54; 5.02) |
| Trabecular area (mm^2^) | -24.30 (-44.76; -3.84) ^a^ | -35.85 (-59.44; -12.26) ^a^ | -23.81 (-44.29; -3.32) ^a^ | -32.34 (-56.28; -8.40) ^a^ | -24.23 (-45.30; -3.16) ^a^ | -32.62 (-57.59; -7.65) ^a^ |
| *Microarchitecture* |  |  |  |  |  |  |
| Trabecular number (mm^-1^) | 0.08 (0.02; 0.13) ^a^ | 0.07 (0.00; 0.13) | 0.08 (0.02; 0.13) ^a^ | 0.06 (-0.01; 0.13) | 0.02 (-0.03; 0.08) | -0.01 (-0.08; 0.05) |
| Trabecular thickness (mm) | 0.00 (0.00; 0.00) | 0.00 (0.00; 0.00) | 0.00 (0.00; 0.00) | 0.00 (0.00; 0.00) | 0.00 (0.00; 0.00) | 0.00 (0.00; 0.01) |
| Trabecular separation (mm) ^†^ | -0.05 (-0.09; -0.01) ^a^ | -0.05 (-0.09; -0.01) ^a^ | -0.05 (-0.09; -0.01) ^a^ | -0.05 (-0.09; 0.00) | -0.02 (-0.06; 0.02) | 0.00 (-0.05; 0.04) |
| Cortical thickness (mm) | 0.07 (0.03; 0.11) ^a^ | 0.09 (0.04; 0.14) ^a^ | 0.07 (0.03; 0.11) ^a^ | 0.08 (0.03; 0.13) ^a^ | 0.03 (-0.01; 0.07) | 0.03 (-0.02; 0.08) |
| Cortical pore volume (mm^3^) ^†^ | 0.01 (-0.06; 0.07) | -0.03 (-0.11; 0.04) | 0.01 (-0.05; 0.07) | -0.02 (-0.09; 0.06) | -0.02 (-0.08; 0.05) | -0.06 (-0.13; 0.02) |
| Cortical porosity (%)^†^ | -0.05 (-0.11; 0.02) | -0.09 (-0.16; -0.02) ^a^ | -0.04 (-0.11; 0.02) | -0.07 (-0.15; 0.00) | -0.03 (-0.10; 0.03) | -0.06 (-0.14; 0.02) |
| Cortical pore diameter (μm) ^†^ | -0.01 (-0.03; 0.01) | 0.00 (-0.02; 0.02) | -0.01 (-0.03; 0.01) | 0.00 (-0.02; 0.02) | 0.00 (-0.02; 0.02) | 0.01 (-0.01; 0.04) |
| *Strength* |  |  |  |  |  |  |
| Stiffness (N/mm) | 6.89 (0.96; 12.82) ^a^ | 7.99 (1.16; 14.82) ^a^ | 7.07 (1.16; 12.99) ^a^ | 8.94 (2.03; 15.86) ^a^ | 1.09 (-4.58; 6.77) | 0.85 (-5.88; 7.57) |
| Failure load (N) | 313.20 (39.84; 586.57) ^a^ | 359.48 (44.32; 674.63) ^a^ | 321.34 (48.33; 594.35) ^a^ | 401.34 (82.27; 720.42) ^a^ | 34.17 (-226.24; 294.57) | 11.98 (-296.60; 320.55) |

Data are shown as beta’s, with 95% CIs.

* Adjusted for age, time gap between visit 1 and HR-pQCT in months, educational level, alcohol use and smoking status

** Adjusted for age, BMI, time gap between visit 1 and HR-pQCT in months, educational level, use of medication that affects bone (glucocorticoids, antidepressants, antipsychotics or neuroleptica), alcohol use, smoking status, use of anti-osteoporotic medication, eGFR, moderate-to-vigorous physical activity, and history of cardiovascular disease.

^†^ Variables were log transformed due to a non-normal distribution. Interpretation of the log transformed variables: prediabetes / T2D is associated with an average change of 100×β% in the bone quality parameter.

BMD, bone mineral density; HA, hydroxyapatite; T2D, type 2 diabetes.

^a^ Indicates statistical significance.

Supplemental Table 3. Linear regression analysis investigating the association between standardized parameters of glycemic control and standardized HR-pQCT parameters

| **Men, radius** | | | | | | | | | | | | | | |
| --- | --- | --- | --- | --- | --- | --- | --- | --- | --- | --- | --- | --- | --- | --- |
| **Grade 1- 3 scans** | **Crude model** | | | | | | | **Adjusted model** | | | | | | |
| *Volumetric BMD* | **HbA1c**  **(n=1400)** | **FPG**  **(n=1400)** | **2h-post**  **(n=1334)** | **SAF**  **(n=1298)** | **IGP**  **(n=1336)** | **CGM-CV**  **(n=240)** | **IS-MI (n=415)** | **HbA1c**  **(n=1400)** | **FPG**  **(n=1400)** | **2h-post**  **(n=1334)** | **SAF**  **(n=1298)** | **IGP**  **(n=1336)** | **CGM-CV**  **(n=240)** | **IS-MI (n=415)** |
| Total BMD (mg HA/cm^3^) | 0.09 (0.03; 0.14) ^a^ | 0.11 (0.06; 0.16) ^a^ | 0.11 (0.05; 0.16) ^a^ | -0.07 (-0.13; -0.01) ^a^ | 0.08 (0.02; 0.14) ^a^ | -0.04 (-0.18; 0.09) | -0.14 (-0.24; -0.05) ^a^ | 0.08 (0.02; 0.13) ^a^ | 0.10 (0.04; 0.15) ^a^ | 0.09 (0.04; 0.15) ^a^ | -0.05 (-0.12; 0.01) | 0.06 (0.00; 0.12) | -0.05 (-0.19; 0.09) | -0.12 (-0.24; -0.01) ^a^ |
| Cortical BMD (mg HA/cm^3^) | 0.04 (-0.01; 0.10) | 0.05 (0.00; 0.10) | 0.08 (0.02; 0.13) ^a^ | -0.02 (-0.08; 0.04) | 0.06 (0.01; 0.12) ^a^ | -0.02 (-0.14; 0.11) | -0.12 (-0.21; -0.02) | 0.05 (-0.01; 0.10) | 0.05 (-0.01; 0.10) | 0.07 (0.02; 0.13) ^a^ | -0.01 (-0.07; 0.06) | 0.06 (0.00; 0.12) | -0.03 (-0.16; 0.10) | -0.08 (-0.19; 0.04) |
| Trabecular BMD (mg HA/cm^3^) | 0.06 (0.01; 0.11) ^a^ | 0.09 (0.04; 0.14) ^a^ | 0.06 (0.01; 0.12) ^a^ | -0.10 (-0.16; -0.04) ^a^ | 0.04 (-0.02; 0.10) | -0.02; -0.16; 0.12) | -0.07 (-0.17; 0.02) | 0.06 (0.00; 0.11) | 0.09 (0.03; 0.14) ^a^ | 0.06 (0.00; 0.12) | -0.09 (-0.15; -0.03) ^a^ | 0.03 (-0.03; 0.09) | -0.01 (-0.16; 0.13) | -0.10 (-0.21; 0.01) |
| *Areal parameters* |  |  |  |  |  |  |  |  |  |  |  |  |  |  |
| Total area (mm^2^) | -0.05 (-0.10; 0.00) | 0.06 (-0.11; -0.01) | -0.08 (-0.13; -0.03) | 0.02 (-0.03; 0.08) | -0.07 (-0.12; -0.01) | 0.02 (-0.13; 0.16) | 0.06 (-0.04; 0.15) | -0.09 (-0.15; -0.03) | -0.10 (-0.15; -0.04) | -0.13 (-0.19; -0.07) | 0.00 (-0.07; 0.06) | -0.10 (-0.17; -0.04) | 0.04 (-0.12; 0.19) | 0.13 (0.02; 0.24) |
| Cortical area (mm^2^) | 0.05 (-0.01; 0.10) | 0.07 (0.01; 0.12) | 0.06 (0.01; 0.12) | -0.08 (-0.13; -0.02) | 0.05 (-0.01; 0.10) | -0.05 (-0.19; 0.08) | -0.15 (-0.24; -0.05) | 0.02 (-0.03; 0.08) | 0.03 (-0.02; 0.09) | 0.04 (-0.02; 0.09) | -0.04 (-0.10; 0.02) | 0.02 (-0.04; 0.08) | -0.03 (-0.17; 0.11) | -0.05 (-0.16; 0.07) |
| Trabecular area (mm^2^) | -0.06 (-0.11; -0.01) | -0.07 (-0.12; -0.02) | -0.09 (-0.15 (-0.04) | 0.03 (-0.02; 0.08) | -0.07 (-0.13; -0.02) | 0.03 (-0.12; 0.17) | 0.09 (0.00; 0.19) | -0.09 (-0.15; -0.04) | -0.10 (-0.15; -0.04) | -0.13 (-0.19; -0.07) | 0.00 (-0.06; 0.07) | -0.10 (-0.16; -0.04) | 0.04 (-0.11; 0.19) | 0.14 (0.02; 0.25) |
| *Microarchitecture* |  |  |  |  |  |  |  |  |  |  |  |  |  |  |
| Trabecular number (mm^-1^) | 0.11 (0.06; 0.16) | 0.15 (0.09; 0.20) | 0.10 (0.05; 0.16) | -0.05 (-0.10; 0.01) | 0.11 (0.06; 0.16) | 0.02 (-0.13; 0.17) | -0.06 (-0.15; 0.03) | 0.04 (-0.01; 0.10) | 0.08 (0.02; 0.13) | 0.04 (-0.02; 0.10) | -0.08 (-0.14; -0.02) | 0.04 (-0.02; 0.10) | 0.09 (-0.07; 0.24) | 0.01 (-0.11; 0.12) |
| Trabecular thickness (mm) | -0.06 (-0.11; 0.00) | -0.04 (-0.10; 0.01) | -0.06 (-0.12; -0.01) | -0.14 (-0.19; -0.08) | -0.08 (-0.13; -0.03) | -0.09 (-0.24; 0.05) | -0.01 (-0.10; 0.08) | 0.04 (-0.02; 0.09) | 0.04 (-0.02; 0.09) | 0.03 (-0.03; 0.09) | -0.03 (-0.10; 0.03) | 0.00 (-0.06; 0.06) | -0.06 (-0.21; 0.08) | -0.13 (-0.24; -0.02) |
| Trabecular separation (mm) ^†^ | -0.09 (-0.14; -0.04) | -0.14 (-0.19; -0.08) | -0.09 (-0.14; -0.04) | 0.07 (0.02; 0.13) | -0.09 (-0.15; -0.04) | -0.01 (-0.16; 0.14) | 0.06 (-0.04; 0.15) | -0.04 (-0.10; 0.01) | -0.09 (-0.14; -0.03) | -0.04 (-0.10; 0.02) | 0.09 (0.03; 0.15) | -0.04 (-0.10; 0.02) | -0.07 (-0.23; 0.08) | 0.01 (-0.10; 0.13) |
| Cortical thickness (mm) | -0.08 (-0.14; -0.03) | -0.13 (-0.18; -0.08) | -0.08 (-0.14; -0.03) | 0.07 (0.02; 0.13) | -0.09 (-0.14; -0.03) | -0.02 (-0.16; 0.13) | -0.15 (-0.25; -0.05) | -0.04 (-0.10; 0.02) | -0.08 (-0.14; -0.03) | -0.04 (-0.10; 0.02) | 0.09 (0.03; 0.15) | -0.04 (-0.10; 0.03) | -0.08 (-0.23; 0.07) | -0.08 (-0.20; 0.03) |
| Cortical pore volume (mm^3^) ^†^ | 0.11 (0.06; 0.16) | 0.11 (0.06; 0.16) | 0.11 (0.05; 0.16) | 0.17 (0.12; 0.23) | 0.10 (0.05; 0.15) | -0.14 (-0.28; 0.00) | -0.14 (-0.24; -0.04) | -0.02 (-0.07; 0.03) | -0.01 (-0.06; 0.05) | -0.04 (-0.10; 0.02) | 0.01 (-0.05; 0.07) | -0.04 (-0.10; 0.02) | -0.16 (-0.29; -0.02) | 0.00 (-0.11; 0.11) |
| Cortical porosity (%)^†^ | 0.10 (0.04; 0.15) | 0.08 (0.03; 0.14) | 0.08 (0.03 –0.14) | 0.21 (0.16; 0.27) | 0.08 (0.03; 0.14) | -0.12 (-0.26; 0.02) | -0.07 (-0.17; 0.03) | -0.03 (-0.08; 0.03) | -0.02 (-0.07; 0.04) | -0.05 (-0.11; 0.00) | 0.03 (-0.03; 0.09) | -0.05 (-0.11; 0.01) | -0.15 (-0.28; -0.02) | 0.02 (-0.09; 0.14) |
| Cortical pore diameter (μm) ^†^ | -0.04 (-0.10; 0.01) | -0.06 (-0.11; -0.01) | -0.06 (-0.11; 0.00) | 0.10 (0.04; 0.15) | -0.06 (-0.12; -0.01) | -0.15 (-0.29; 0.00) | 0.00 (-0.10; 0.10) | -0.04 (-0.10; 0.02) | -0.05 (-0.10; 0.01) | -0.06 (-0.12; 0.00) | 0.06 (-0.01; 0.12) | -0.08 (-0.14; -0.02) | -0.19 (-0.34; -0.04) | -0.04 (-0.16; 0.08) |
| *Strength* |  |  |  |  |  |  |  |  |  |  |  |  |  |  |
| Stiffness (N/mm) | -0.02 (-0.07; 0.03) | 0.00 (-0.05 -0.05) | -0.03 (-0.09; 0.02) | -0.14 (-0.20; -0.09) | -0.05 (-0.10; 0.01) | -0.09 (-0.23; 0.06) | -0.07 (-0.15; 0.03) | 0.01 (-0.05; 0.06) | 0.02 (-0.04; 0.07) | -0.01 (-0.07; 0.05) | -0.05 (-0.11; 0.02) | -0.02 (-0.08; 0.04) | -0.04 (-0.19; 0.11) | -0.04 (-0.14; 0.07) |
| Failure load (N) | -0.02 (-0.07; 0.03) | 0.00 (-0.05; 0.05) | -0.03 (-0.09; 0.02) | -0.14 (-0.19; -0.09) | -0.05 (-0.10; 0.01) | -0.08 (-0.23; 0.07) | -0.06 (-0.14; 0.03) | 0.01 (-0.05; 0.06) | 0.01 (-0.04; 0.07) | -0.01 (-0.07; 0.05) | -0.05 (-0.11; 0.02) | -0.02 (-0.08; 0.04) | -0.03 (-0.18; 0.12) | -0.03 (-0.13; 0.08) |
| **Men, tibia** | | | | | | | | | | | | | | |
| **Grade 1- 3 scans** | **Crude model** | | | | | | | **Adjusted model** | | | | | | |
| *Volumetric BMD* | **HbA1c**  **(n=946)** | **FPG**  **(n=946)** | **2h-post**  **(n=911)** | **SAF**  **(n=908)** | **IGP**  **(n=914)** | **CGM-CV**  **(n=190)** | **IS-MI (n=391)** | **HbA1c**  **(n=946)** | **FPG**  **(n=946)** | **2h-post**  **(n=911)** | **SAF**  **(n=908)** | **IGP**  **(n=914)** | **CGM-CV**  **(n=190)** | **IS-MI (n=391)** |
| Total BMD (mg HA/cm^3^) | 0.02 (-0.05; 0.08) | 0.10 (0.03; 0.17) ^a^ | 0.09 (0.03; 0.16) ^a^ | -0.06 (-0.13; 0.02) | 0.07 (0.00; 0.14) | -0.03 (-0.18; 0.12) | -0.06 (-0.16; 0.05) | 0.06 (-0.01; 0.13) | 0.09 (0.03; 0.16) ^a^ | 0.09 (0.02; 0.16) ^a^ | -0.02 (-0.09; 0.05) | 0.06 (-0.01; 0.14) | 0.00 (-0.16; 0.16) | -0.0 (-0.15; 0.08) |
| Cortical BMD (mg HA/cm^3^) | 0.04 (-0.03; 0.10) | 0.07 (0.01; 0.13) ^a^ | 0.06 (0.00; 0.13) | -0.04 (-0.11; 0.03) | 0.04 (-0.03; 0.11) | -0.04 (-0.19; 0.11) | -0.05 (-0.15; 0.05) | 0.03 (-0.04; 0.09) | 0.06 (-0.01; 0.13) | 0.06 (-0.01; 0.13) | -0.03 (-0.10; 0.04) | 0.03 (-0.04; 0.10) | -0.02 (-0.18; 0.13) | -0.09 (-0.21; 0.02) |
| Trabecular BMD (mg HA/cm^3^) | 0.05 (-0.02; 0.11) | 0.05 (-0.01; 0.12) | 0.05 (-0.02; 0.12) | -0.06 (-0.13; 0.01) | 0.04 (-0.03; 0.11) | 0.02 (-0.15; 0.17) | 0.01 (-0.08; 0.11) | 0.06 (-0.01; 0.12) | 0.06 (-0.01; 0.13) | 0.06 (-0.01; 0.13) | -0.02 (-0.09; 0.06) | 0.05 (-0.02; 0.12) | 0.05 (-0.11; 0.21) | 0.02 (-0.09; 0.14) |
| *Areal parameters* |  |  |  |  |  |  |  |  |  |  |  |  |  |  |
| Total area (mm^2^) | -0.07 (-0.14; -0.01) | -0.11 (-0.17; -0.04) | -0.12 (-0.19; -0.06) | -0.05 (-0.11; 0.02) | -0.06 (-0.13; 0.01) | -0.01 (-0.17; 0.16) | 0.04 (-0.05; 0.15) | -0.10 (-0.17; - 0.03) | -0.14 (-0.21; -0.07) | -0.17 (-0.24; -0.10) | -0.03 (-0.11; 0.04) | -0.12 (-0.20; -0.05) | 0.03 (-0.14; 0.21) | 0.12 (0.01; 0.23) |
| Cortical area (mm^2^) | -0.03 (-0.09; 0.04) | 0.00 (-0.06; 0.07) | -0.03 (-0.09 -0.04) | -0.16 (-0.22; -0.09) | 0.04 (-0.10; 0.03) | -0.12 (-0.27; 0.03) | -0.04 (-0.15; 0.06) | -0.01 (-0.08; 0.05) | 0.01 (-0.06; 0.08) | -0.01 (-0.08; 0.06) | -0.05 (-0.13; 0.02) | -0.02 (-0.09; 0.05) | -0.05 (-0.21; 0.10) | 0.02 (-0.09; 0.14) |
| Trabecular area (mm^2^) | -0.07 (-0.13; -0.01) | -0.11 (-0.17; -0.04) | -0.11 (-0.18; -0.05) | -0.02 (-0.08 -0.05) | -0.05 (-0.12; 0.01) | 0.02 (-0.15; 0.18) | 0.05 (-0.05; 0.15) | -0.09 (-0.16; -0.02) | -0.14 (-0.21; -0.07) | -0.16 (-0.23; -0.09) | -0.02 (-0.10; 0.05) | -0.11 (-0.19; -0.04) | 0.04 (-0.13; 0.22) | 0.11 (-0.01; 0.22) |
| *Microarchitecture* |  |  |  |  |  |  |  |  |  |  |  |  |  |  |
| Trabecular number (mm^-1^) | 0.05 (-0.01; 0.12) | 0.07 (0.01; 0.14) | 0.07 (0.00; 0.13) | -0.10 (-0.16; -0.03) | 0.10 (0.04; 0.16) | -0.12 (-0.27; 0.04) | -0.04 (-0.14; 0.06) | 0.01 (-0.06; 0.07) | 0.01 (-0.05; 0.08) | 0.03 (-0.04; 0.10) | -0.01 (-0.09; 0.06) | 0.04 (-0.03; 0.11) | -0.05 (-0.21; 0.11) | 0.08 (-0.03; 0.19) |
| Trabecular thickness (mm) | -0.01 (-0.08; 0.05) | -0.02 (-0.08; 0.05) | -0.04 (-0.11; 0.03) | -0.06 (-0.12; 0.01) | -0.07 (-0.13; 0.00) | 0.10 (-0.04; 0.24) | 0.09 (-0.01; 0.19) | 0.07 (0.01; 0.14) | 0.07 (0.01; 0.14) | 0.06 (-0.02; 0.13) | 0.00 (-0.08; 0.07) | 0.03 (-0.05; 0.10) | 0.11 (-0.04; 0.26) | -0.03 (-0.14; 0.09) |
| Trabecular separation (mm) ^†^ | -0.04 (-0.11; 0.02) | -0.07 (-0.13; 0.00) | -0.06 (-0.12; 0.01) | 0.11 (0.04; 0.17) | -0.09 (-0.15; -0.02) | 0.10 (-0.06; 0.26) | 0.03 (-0.06; 0.13) | -0.01 (-0.08; 0.06) | -0.02 (-0.09; 0.05) | -0.03 (-0.10; 0.04) | 0.01 (-0.06; 0.08) | -0.04 (-0.11; 0.03) | 0.03 (-0.13; 0.19) | -0.06 (-0.17; 0.05) |
| Cortical thickness (mm) | 0.00 (-0.07; 0.06) | 0.04 (-0.03; 0.10) | 0.02 (-0.05; 0.08) | -0.13 (-0.20; -0.07) | -0.01 (-0.08; 0.06) | -0.10 (-0.24; 0.05) | -0.06 (-0.16; 0.05) | 0.02 (-0.05; 0.09) | 0.06 (-0.01; 0.13) | 0.05 (-0.02; 0.12) | -0.04 (-0.11; 0.04) | 0.03 (-0.05; 0.10) | -0.05 (-0.20; 0.11) | -0.03 (-0.15; 0.10) |
| Cortical pore volume (mm^3^) ^†^ | 0.09 (0.03; 0.15) | 0.07 (0.00; 0.13) | 0.07 (0.00; 0.13) | 0.07 (0.00; 0.13) | 0.10 (0.03; 0.16) | 0.05 (-0.10; 0.21) | -0.01 (-0.10; 0.09) | 0.02 (-0.05; 0.09) | -0.01 (-0.08; 0.06) | -0.02 (-0.09; 0.05) | 0.00 (-0.07; 0.07) | 0.00 (-0.07; 0.07) | 0.05 (-0.11; 0.21) | 0.10 (-0.01; 0.21) |
| Cortical porosity (%)^†^ | 0.10 (0.03; 0.16) | 0.06 (0.00; 0.13) | 0.08 (0.02; 0.15) | 0.15 (0.09; 0.21) | 0.11 (0.05; 0.18) | 0.12 (-0.03; 0.28) | 0.04 (-0.05; 0.14) | 0.02 (-0.04; 0.09) | -0.02 (-0.08; 0.05) | -0.01 (-0.08; 0.06) | 0.03 (-0.04; 0.10) | 0.01 (-0.06; 0.08) | 0.09 (-0.07; 0.24) | 0.10 (-0.01; 0.21) |
| Cortical pore diameter (μm) ^†^ | 0.00 (-0.07; 0.06) | -0.05 (-0.12; 0.01) | -0.09 (-0.15; -0.02) | 0.05 (-0.02; 0.11) | -0.10 (-0.17; -0.04) | 0.20 (0.06; 0.35) | 0.10 (0.00; 0.20) | 0.09 (0.03; 0.16) | 0.05 (-0.02 -0.12) | 0.01 (-0.06; 0.08) | 0.05 (-0.03; 0.12) | 0.01 (-0.06; 0.08) | 0.18 (0.04; 0.33) | -0.04 (-0.15; 0.07) |
| *Strength* |  |  |  |  |  |  |  |  |  |  |  |  |  |  |
| Stiffness (N/mm) | -0.05 (-0.11; 0.01) | -0.05 (-0.12; 0.01) | -0.08 (-0.15; -0.02) | -0.18 (-0.24; -0.11) | -0.06 (-0.13; 0.00) | -0.05 (-0.21; 0.11) | 0.02 (-0.08; 0.12) | -0.01 (-0.08; 0.06) | -0.03 (-0.09; 0.04) | -0.05 (-0.12; 0.02) | -0.04 (-0.12; 0.03) | -0.04 (-0.11; 0.03) | 0.04 (-0.12; 0.20) | 0.06 (-0.05; 0.16) |
| Failure load (N) | -0.05 (-0.11; 0.02) | -0.05 (-0.11; 0.01) | -0.08 (-0.14; -0.01) | -0.18 (-0.24; -0.11) | -0.06 (-0.12; 0.01) | -0.06 (-0.22; 0.10) | 0.02 (-0.08; 0.11) | -0.01 (-0.08; 0.05) | -0.03 (-0.10; 0.04) | -0.05 (-0.12; 0.02) | -0.05 (-0.12; 0.03) | -0.04 (-0.11; 0.03) | 0.03 (-0.13; 0.20) | 0.06 (-0.04; 0.17) |
| **Women, radius** | | | | | | | | | | | | | | |
| **Grade 1- 3 scans** | **Crude model** | | | | | | | **Adjusted model** | | | | | | |
| *Volumetric BMD* | **HbA1c**  **(n=1415)** | **FPG**  **(n=1415)** | **2h-post**  **(n=1383)** | **SAF**  **(n=1320)** | **IGP**  **(n=1384)** | **CGM-CV**  **(n=221)** | **IS-MI (n=372)** | **HbA1c**  **(n=1415)** | **FPG**  **(n=1415)** | **2h-post**  **(n=1383)** | **SAF**  **(n=1320)** | **IGP**  **(n=1384)** | **CGM-CV**  **(n=221)** | **IS-MI (n=372)** |
| Total BMD (mg HA/cm^3^) | 0.15 (0.10; 0.20) ^a^ | 0.16 (0.11; 0.21) | 0.20 (0.15; 0.25) ^a^ | -0.01 (-0.06; 0.05) | 0.17 (0.11; 0.22) ^a^ | 0.14 (0.00; 0.27) | -0.21 (-0.30; 0.11) | 0.07 (0.02; 0.13) | 0.08 (0.02; 0.13) ^a^ | 0.13 (0.08; 0.18) ^a^ | -0.02 (-0.07; 0.04) | 0.11 (0.05; 0.16) ^a^ | 0.16 (0.02; 0.30) ^a^ | -0.12 (-0.23; -0.01) ^a^ |
| Cortical BMD (mg HA/cm^3^) | 0.09 (0.05; 0.14) | 0.10 (0.05; 0.14) ^a^ | 0.13 (0.09; 0.18) ^a^ | -0.04 (-0.08; 0.01) | 0.12 (0.07; 0.17) ^a^ | 0.03 (-0.09; 0.15) | -0.14 (-0.23; -0.05) ^a^ | 0.04 (-0.01; 0.09) | 0.04 (-0.01; 0.09) | 0.03 (0.04; 0.14) ^a^ | -0.05 (-0.10; 0.00) | 0.09 (0.04; 0.13) ^a^ | 0.04 (-0.08; 0.16) | -0.09 (-0.19; 0.02) |
| Trabecular BMD (mg HA/cm^3^) | 0.15 (0.09; 0.20) ^a^ | 0.17 (0.11; 0.22) ^a^ | 0.16 (0.11; 0.21) ^a^ | 0.00 (-0.06; 0.06) | 0.13 (0.07; 0.18) ^a^ | 0.14 (-0.01; 0.29) | -0.13 (-0.22; -0.03) ^a^ | 0.10 (0.04; 0.15) ^a^ | 0.10 (0.05; 0.16) ^a^ | 0.11 (0.06; 0.17) ^a^ | 0.00 (-0.06; 0.06) | 0.09 (0.03; 0.14) ^a^ | 0.17 (0.02; 0.33) | -0.05 (-0.16; 0.06) |
| *Areal parameters* |  |  |  |  |  |  |  |  |  |  |  |  |  |  |
| Total area (mm^2^) | -0.02 (-0.07; 0.03) | -0.02 (-0.07; 0.04) | -0.10 (-0.16; -0.05) | -0.01 (-0.06; 0.05) | -0.10 (-0.15; -0.05) | -0.21 (-0.36; -0.07) | 0.10 (0.00; 0.21) | -0.03 (-0.09; 0.03) | -0.03 (-0.08; 0.03) | -0.12 (-0.18; -0.06) | -0.02 (-0.08; 0.04) | -0.12 (-0.18; -0.06) | -0.20 (-0.35; -0.04) | 0.11 (-0.01; 0.22) |
| Cortical area (mm^2^) | 0.03 (-0.02; 0.08) | 0.07 (0.01; 0.12) | 0.04 (-0.02; 0.09) | -0.17 (-0.22; -0.12) | 0.00 (-0.05; 0.05) | -0.06 (-0.20; 0.08) | -0.14 (-0.25; -0.04) | 0.05 (0.00; 0.10) | 0.05 (0.00; 0.10) | 0.06 (0.01; 0.11) | -0.05 (-0.10; 0.00) | 0.04 (-0.01; 0.10) | 0.04 (-0.10; 0.17) | -0.09 (-0.20; 0.02) |
| Trabecular area (mm^2^) | -0.03 (-0.08; 0.02) | -0.03 (-0.09; 0.02) | -0.11 (-0.16; -0.06) | 0.02 (-0.03; 0.08) | -0.10 (-0.15; -0.05) | -0.20 (-0.34; -0.05) | 0.13 (0.03; 0.23) | -0.04 (-0.10; 0.02) | -0.03 (-0.09; 0.02) | -0.13 (-0.18; -0.07) | -0.01 (-0.07; 0.05) | -0.12 (-0.18; -0.06) | -0.20 (-0.35; -0.05) | 0.12 (0.01; 0.23) |
| *Microarchitecture* |  |  |  |  |  |  |  |  |  |  |  |  |  |  |
| Trabecular number (mm^-1^) | 0.09 (0.04; 0.14) | 0.13 (0.08; 0.18) | 0.11 (0.06; 0.16) | -0.07 (-0.12; -0.02) | 0.06 (0.01; 0.11) | 0.05 (-0.10; 0.20) | -0.07 (-0.17; 0.03) | 0.04 (-0.01; 0.10) | 0.06 (0.01; 0.12) | 0.07 (0.01; 0.12) | -0.05 (-0.11; 0.01) | 0.04 (-0.02; 0.09) | 0.10 (-0.04; 0.25) | 0.04 (-0.08; 0.15) |
| Trabecular thickness (mm) | 0.04 (-0.01; 0.09) | 0.04 (-0.01; 0.10) | 0.04 (-0.01; 0.10) | -0.03 (-0.09; 0.02) | 0.03 (-0.02; 0.09) | 0.10 (-0.06; 0.26) | -0.10 (-0.20; -0.01) | 0.09 (0.04; 0.15) | 0.08 (0.02; 0.14) | 0.09 (0.03; 0.15) | 0.03 (-0.03; 0.09) | 0.09 (0.04; 0.15) | 0.13 (-0.03; 0.30) | -0.12 (-0.23; -0.01) |
| Trabecular separation (mm) ^†^ | -0.09 (-0.14; -0.03) | -0.12 (-0.18; -0.07) | -0.10 (-0.16; -0.05) | 0.07 (0.02; 0.13) | -0.06 (-0.11; 0.00) | -0.05 (-0.20; 0.11) | 0.07 (-0.03; 0.18) | -0.05 (-0.11; 0.01) | -0.07 (-0.13 --0.02) | -0.08 (-0.13; -0.02) | 0.04 (-0.01; 0.10) | -0.04 (-0.10; 0.02) | -0.10 (-0.25; 0.05) | -0.03 (-0.14; 0.08) |
| Cortical thickness (mm) | 0.03 (-0.02; 0.08) | 0.06 (0.01; 0.11) | 0.06 (0.01; 0.11) | -0.16 (-0.21; -0.11) | 0.02 (-0.03 0.08) | 0.00 (-0.14; 0.14) | -0.16 (-0.27; -0.06) | 0.05 (0.00; 0.11) | 0.05 (0.00; 0.10) | 0.09 (0.04; 0.14) | -0.04 (-0.09; 0.01) | 0.07 (0.02; 0.12) | 0.09 (-0.04; 0.21) | -0.11 (-0.22; -0.01) |
| Cortical pore volume (mm^3^) ^†^ | 0.22 (0.16; 0.27) | 0.17 (0.12; 0.22) | 0.14 (0.09; 0.20) | 0.22 (0.17; 0.27) | 0.15 (0.09; 0.20) | 0.13 (-0.01; 0.27) | -0.08 (-0.18; 0.01) | 0.06 (0.01; 0.11) | 0.04 (-0.01; 0.09) | -0.01 (-0.06 -0.04) | 0.03 (-0.02; 0.08) | -0.03 (-0.08; 0.02) | 0.05 (-0.07; 0.18) | 0.00 (-0.10; 0.10) |
| Cortical porosity (%)^†^ | 0.19 (0.13; 0.24) | 0.13 (0.08; 0.18) | 0.12 (0.07; 0.17) | 0.26 (0.21; 0.32) | 0.14 (0.09; 0.19) | 0.15 (0.01; 0.29) | -0.03 (-0.12; 0.07) | 0.04 (-0.01; 0.08) | 0.02 (-0.03; 0.07) | -0.03 (-0.07; 0.02) | 0.05 (0.00; 0.09) | -0.04 (-0.09; 0.01) | 0.05 (-0.07; 0.16) | 0.03 (-0.06; 0.12) |
| Cortical pore diameter (μm) ^†^ | 0.12 (0.07; 0.17) | 0.08 (0.03; 0.14) | 0.11 (0.05; 0.16) | 0.16 (0.10; 0.21) | 0.11 (0.06; 0.16) | 0.08 (-0.07; 0.24) | -0.05 (-0.15; 0.05) | 0.05 (-0.01; 0.11) | 0.04 (-0.01; 0.10) | 0.05 (-0.01; 0.10) | 0.04 (-0.02; 0.09) | 0.04 (-0.02; 0.09) | -0.03 (-0.18; 0.13) | -0.01 (-0.12; 0.10) |
| *Strength* |  |  |  |  |  |  |  |  |  |  |  |  |  |  |
| Stiffness (N/mm) | 0.03 (-0.02; 0.09) | 0.08 (0.02; 0.13) | 0.01 (-0.04; 0.07) | -0.16 (-0.21; -0.10) | -0.03 (-0.08; 0.02) | -0.03 (-0.18; 0.12) | -0.10 (-0.20; 0.00) | 0.09 (0.03; 0.14) | 0.09 (0.04; 0.14) | 0.06 (0.01; 0.11) | -0.03 (-0.09; 0.02) | 0.04 (-0.01; 0.10) | 0.08 (-0.07; 0.22) | -0.07 (-0.17; 0.04) |
| Failure load (N) | 0.04 (-0.02; 0.09) | 0.08 (0.03; 0.13) | 0.01 (-0.04; 0.07) | -0.16 (-0.21; -0.11) | -0.03 (-0.09; 0.02) | -0.03 (-0.19; 0.12) | -0.10 (-0.20; 0.00) | 0.09 (0.03; 0.14) | 0.09 (0.04; 0.14) | 0.06 (0.00; 0.11) | -0.04 (-0.09; 0.02) | 0.04 (-0.01; 0.09) | 0.08 (-0.07; 0.22) | -0.06 (-0.17; 0.04) |
| **Women, tibia** | | | | | | | | | | | | | | |
| **Grade 1- 3 scans** | **Crude model** | | | | | | | **Adjusted model** | | | | | | |
| *Volumetric BMD* | **HbA1c**  **(n=969)** | **FPG**  **(n=969)** | **2h-post**  **(n=947)** | **Mean AFR**  **(n=944)** | **IGP**  **(n=948)** | **CGM-CV**  **(n=197)** | **IS-MI (n=371)** | **HbA1c**  **(n=969)** | **FPG**  **(n=969)** | **2h-post**  **(n=947)** | **Mean AFR**  **(n=944)** | **IGP**  **(n=948)** | **CGM-CV**  **(n=197)** | **IS-MI (n=371)** |
| Total BMD (mg HA/cm^3^) | 0.13 (0.07; 0.19) ^a^ | 0.11 (0.05; 0.18) ^a^ | 0.16 (0.10; 0.22) ^a^ | 0.02 (-0.04; 0.09) | 0.13 (0.06; 0.19) ^a^ | 0.04 (-0.12; 0.19) | -0.16 (-0.25; -0.07) ^a^ | 0.08 (0.01; 0.14) | 0.04 (-0.02; 0.10) | 0.09 (0.03; 0.16) ^a^ | 0.03 (-0.04; 0.09) | 0.07 (0.00; 0.13) | 0.05 (-0.11; 0.20) | -0.05 (-0.15; 0.06) |
| Cortical BMD (mg HA/cm^3^) | 0.05 (0.00; 0.10) | 0.05 (0.00; 0.10) | 0.10 (0.05; 0.15) ^a^ | -0.02 (-0.07; 0.03) | 0.10 (0.04; 0.15) ^a^ | 0.00 (-0.11; 0.11) | -0.11 (-0.19; -0.03) ^a^ | 0.02 (-0.03; 0.07) | 0.02 (-0.03; 0.07) | 0.06 (0.01; 0.11) ^a^ | -0.03(-0.08; 0.03) | 0.07 (0.02; 0.12) ^a^ | -0.02 (-0.13; 0.10) | -0.05 (-0.14; 0.04) |
| Trabecular BMD (mg HA/cm^3^) | 0.07 (0.01; 0.14) ^a^ | 0.07 (0.00; 0.13) | 0.09 (0.02; 0.15) ^a^ | -0.01 (-0.08; 0.06) | 0.05 (-0.01; 0.12) | 0.04 (-0.12; 0.20) | -0.10 (-0.20; 0.00) | 0.03 (-0.03; 0.10) | 0.01 (-0.06; 0.07) | 0.03 (-0.03; 0.10) | 0.00 (-0.07; 0.07) | 0.01 (-0.06; 0.08) | 0.07 (-0.09; 0.23) | -0.01 (-0.12; 0.11) |
| *Areal parameters* |  |  |  |  |  |  |  |  |  |  |  |  |  |  |
| Total area (mm^2^) | -0.06 (-0.13; 0.00) | -0.04 (-0.10; 0.03) | -0.10 (-0.16; -0.03) | -0.06 (-0.13; 0.00) | -0.10 (-0.16; -0.03) | -0.13 (-0.28 -0.03) | 0.03 (-0.07; 0.13) | -0.09 (-0.16; -0.02) | -0.06 (-0.13; 0.01) | -0.12 (-0.19; -0.05) | -0.09 (-0.16; -0.02) | -0.12 (-0.19; -0.05) | -0.12 (-0.28; 0.04) | 0.05 (-0.06; 0.17) |
| Cortical area (mm^2^) | -0.05 (-0.11; 0.02) | -0.02 (-0.08; 0.04) | -0.01 (-0.08; 0.05) | -0.19 (-0.25; -0.13) | -0.06 (-0.12; 0.01) | -0.11 (-0.26; 0.04) | -0.11 (-0.21; -0.01) | 0.04 (-0.02; 0.09) | 0.01 (-0.04; 0.07) | 0.03 (-0.03; 0.09) | -0.01 (-0.07; 0.05) | 0.02 (-0.04; 0.08) | -0.05 (-0.17; 0.08) | -0.03 (-0.12; 0.07) |
| Trabecular area (mm^2^) | -0.06 (-0.12; 0.01) | -0.04 (-0.10; 0.03) | -0.09 (-0.16; -0.03) | -0.03 (-0.09; 0.03) | -0.09 (-0.15; -0.02) | -0.10 (-0.25; 0.05) | 0.05 (-0.05; 0.15) | -0.10 (-0.17; -0.03) | -0.06 (-0.13; 0.01) | -0.12 (-0.19; -0.05) | -0.09 (-0.15; -0.02) | -0.12 (-0.19; -0.05) | -0.10 (-0.26; 0.06) | 0.06 (-0.06; 0.18) |
| *Microarchitecture* |  |  |  |  |  |  |  |  |  |  |  |  |  |  |
| Trabecular number (mm^-1^) | 0.02 (-0.04; 0.09) | 0.01 (-0.05; 0.08) | 0.04 (-0.02; 0.11) | -0.10 (-0.16; -0.03) | 0.04 (-0.03; 0.10) | -0.08 (-0.23; 0.06) | -0.14 (-0.24; -0.03) | 0.00 (-0.07; 0.07) | -0.03 (-0.10; 0.03) | 0.00 (-0.07; 0.07) | -0.07 (-0.13; 0.00) | 0.01 (-0.06; 0.08) | -0.05 (-0.20; 0.09) | -0.02 (-0.13; 0.10) |
| Trabecular thickness (mm) | 0.03 (-0.03; 0.10) | 0.04 (-0.03; 0.10) | 0.03 (-0.03; 0.10) | 0.05 (-0.02; 0.11) | 0.00 (-0.07; 0.06) | 0.12 (-0.03; 0.26) | 0.05 (-0.06; 0.16) | 0.05 (-0.02; 0.12) | 0.04 (-0.03; 0.11) | 0.05 (-0.03; 0.12) | 0.07 (0.00; 0.14) | 0.00 (-0.07; 0.07) | 0.14 (-0.01; 0.29) | 0.02 (-0.10; 0.15) |
| Trabecular separation (mm) ^†^ | -0.03 (-0.09; 0.04) | -0.02 (-0.08; 0.04) | -0.05 (-0.11; 0.02) | 0.09 (0.03; 0.15) | -0.03 (-0.10; 0.03) | 0.09 (-0.05; 0.23) | 0.14 (0.03; 0.25) | -0.01 (-0.08; 0.06) | 0.02 (-0.04; 0.09) | -0.01 (-0.08; 0.06) | 0.06 (-0.01; 0.12) | -0.01 (-0.08; 0.06) | 0.06 (-0.08; 0.20) | 0.02 (-0.10; 0.13) |
| Cortical thickness (mm) | -0.03 (-0.09; 0.04) | -0.01 (-0.07; 0.06) | 0.02 (-0.05; 0.08) | -0.15 (-0.21; 0.09) | -0.03 (-0.09; 0.04) | -0.07 (-0.22; 0.09) | -0.11 (-0.21; -0.01) | 0.06 (0.00; 0.12) | 0.03 (-0.03; 0.09) | 0.06 (0.00; 0.12) | 0.02 (-0.04; 0.08) | 0.05 (-0.01; 0.11) | -0.01 (-0.15; 0.13) | -0.04 (-0.14; 0.07) |
| Cortical pore volume (mm^3^) ^†^ | 0.20 (0.14; 0.26) | 0.16 (0.10; 0.22) | 0.13 (0.06; 0.19) | 0.19 (0.13; 0.26) | 0.15 (0.08; 0.21) | 0.04 (-0.12; 0.20) | -0.06 (-0.15; 0.04) | 0.02 (-0.04; 0.08) | -0.01 (-0.06; 0.05) | -0.04 (-0.10; 0.02) | 0.04 (-0.02; 0.10) | -0.06 (-0.13; 0.00) | 0.02 (-0.12; 0.17) | 0.02 (-0.08; 0.12) |
| Cortical porosity (%)^†^ | 0.19 (0.13; 0.25) | 0.14 (0.08; 0.20) | 0.11 (0.05; 0.18) | 0.26 (0.20; 0.32) | 0.15 (0.08; 0.21) | 0.09 (-0.07; 0.24) | 0.01 (-0.08; 0.10) | 0.00 (-0.06; 0.05) | -0.01 (-0.07; 0.04) | -0.05 (-0.10; 0.01) | 0.05 (0.00; 0.10) | -0.07 (-0.12; -0.01) | 0.05 (-0.08; 0.18) | 0.04 (-0.05; 0.12) |
| Cortical pore diameter (μm) ^†^ | 0.00 (-0.06; 0.07) | 0.00 (-0.06; 0.07) | -0.01 (-0.08; 0.05) | 0.14 (0.08; 0.21) | -0.05 (-0.12; 0.01) | 0.02 (-0.13; 0.17) | 0.08 (-0.02; 0.18) | 0.02 (-0.05; 0.09) | 0.03 (-0.04; 0.10) | 0.02 (-0.05; 0.09) | 0.13 (0.07; 0.20) | -0.04 (-0.11; 0.03) | -0.01 (-0.16; 0.14) | 0.00 (-0.12; 0.11) |
| *Strength* |  |  |  |  |  |  |  |  |  |  |  |  |  |  |
| Stiffness (N/mm) | -0.04 (-0.10; 0.03) | 0.00 (-0.07; 0.06) | -0.02 (-0.09; 0.04) | -0.18 (-0.24; -0.12) | -0.08 (-0.14; -0.02) | -0.08 (-0.24; 0.08) | -0.09 (-0.19; 0.02) | 0.02 (-0.04; 0.08) | 0.01 (-0.05; 0.07) | -0.01 (-0.07; 0.06) | -0.05 (-0.11; 0.01) | -0.03 (-0.09; 0.03) | -0.02 (-0.16; 0.13) | 0.00 (-0.10; 0.11) |
| Failure load (N) | -0.04 (-0.10; 0.02) | 0.00 (-0.07; 0.06) | -0.03 (-0.09; 0.04 | -0.19 (-0.25; -0.13) | -0.08 (-0.14; -0.01) | -0.09 (-0.25; 0.07) | -0.09 (-0.19; 0.01) | 0.01 (-0.05; 0.07) | 0.00 (-0.06; 0.06) | -0.01 (-0.07; 0.05) | -0.06 (-0.12; 0.00) | -0.03 (-0.09; 0.03) | -0.03 (-0.17; 0.11) | 0.01 (-0.10; 0.11) |

Data are shown as beta’s, with 95% CIs.

* Adjusted for age, BMI, time gap between visit 1 and HR-pQCT in months, educational level, use of medication that affects bone (glucocorticoids, antidepressants, antipsychotics or neuroleptica), alcohol use, smoking status, use of anti-osteoporotic medication, eGFR, moderate-to-vigorous physical activity, and history of cardiovascular disease.

^†^ Interpretation of the log transformed variables: prediabetes / T2D is associated with an average change of 100×β% in the bone quality parameter.

95% CI, 95% confidence interval; 2h-post, plasma glucose two hours post glucose load; BMD, bone mineral density; CGM- CV, continuous glucose monitoring assessed coefficient of variation; FPG, fasting plasma glucose, HA, hydroxyapatite; HbA1c, glycated hemoglobin; IGP, incremental glucose peak; IS-MI, insulin sensitivity measured as the Matsuda index; SAF, skin autofluorescence; T2D, type 2 diabetes.
